# High-Throughput Detection of Induced Mutations and Natural Variation Using KeyPoint™ Technology

**DOI:** 10.1371/journal.pone.0004761

**Published:** 2009-03-13

**Authors:** Diana Rigola, Jan van Oeveren, Antoine Janssen, Anita Bonné, Harrie Schneiders, Hein J. A. van der Poel, Nathalie J. van Orsouw, René C. J. Hogers, Michiel T. J. de Both, Michiel J. T. van Eijk

**Affiliations:** Keygene NV, Wageningen, The Netherlands; Cinvestav, Mexico

## Abstract

Reverse genetics approaches rely on the detection of sequence alterations in target genes to identify allelic variants among mutant or natural populations. Current (pre-) screening methods such as TILLING and EcoTILLING are based on the detection of single base mismatches in heteroduplexes using endonucleases such as CEL 1. However, there are drawbacks in the use of endonucleases due to their relatively poor cleavage efficiency and exonuclease activity. Moreover, pre-screening methods do not reveal information about the nature of sequence changes and their possible impact on gene function. We present KeyPoint™ technology, a high-throughput mutation/polymorphism discovery technique based on massive parallel sequencing of target genes amplified from mutant or natural populations. KeyPoint combines multi-dimensional pooling of large numbers of individual DNA samples and the use of sample identification tags (“sample barcoding”) with next-generation sequencing technology. We show the power of KeyPoint by identifying two mutants in the tomato eIF4E gene based on screening more than 3000 M2 families in a single GS FLX sequencing run, and discovery of six haplotypes of tomato eIF4E gene by re-sequencing three amplicons in a subset of 92 tomato lines from the EU-SOL core collection. We propose KeyPoint technology as a broadly applicable amplicon sequencing approach to screen mutant populations or germplasm collections for identification of (novel) allelic variation in a high-throughput fashion.

## Introduction

Rapid, high-throughput mutation and single nucleotide polymorphism discovery technologies are fundamental to identify allelic variants in large populations. Such analyses are very useful for functional genetics, clinical diagnostics, forensic medicine, population genetics, molecular epidemiology, plant and animal breeding. Mutations are the basis of genetic variation and mutant populations are indispensable genetic resources in all organisms. This variation can be either naturally occurring or, in plants, animals and lower organisms, induced by chemical or physical treatments. Mutation induction, for example, has played an important role in the genetic improvement of crop species that are of economic importance [1].

Although sequencing is considered the “golden standard” for DNA-based mutation detection because it reveals the exact location and the type of mutations, direct gene sequencing methods have rarely been used to screen large (mutant) populations because of cost limitations. Instead, in the past a number of prescreening methods have been developed and applied in order to scan amplicons in large (mutant) populations for the presence of sequence polymorphisms, prior to confirmation by Sanger sequencing. For example, the characteristic DNA properties of melting temperature and single strand conformation have been used in techniques such as Denaturing High Performance Liquid Chromatograpy (DHPLC), Denaturing Gradient Gel Electrophoresis (DGGE), Temperature Gradient Gel Electrophoresis (TGGE), Single Strand Conformational Polymorphism analysis (SSCP) [reviewed in 2], and High Resolution DNA Melting (HRM) [3]. In addition, other procedures have been developed including the Protein Truncation Test [4], enzymatic or chemical cleavage methods [reviewed in 5], the Restriction Site Mutation (RSM) method [6] and s-RT-MELT technology [7].

The Targeting Induced Local Lesions In Genomes (TILLING) technique makes use of chemical mutagenesis to induce mutations throughout an entire genome and applies an enzyme-mediated detection method (e.g. based on CEL I nuclease) combined with DHPLC or gel electrophoresis detection [8], [9], [10]. A variation of the TILLING method, known as EcoTILLING [11], aims at the detection of natural (allelic) variation in the germplasm. TILLING has been successfully applied to organisms such as zebrafish [12], *C. elegans* [13], and plants including *Arabidopsis* [14], rice [15], soybean [16] wheat [17] and maize [18]. However, limitations of an endonuclease such as CEL I are its relatively poor cleavage efficiency and 5′-3′ exonuclease activity. This diminishes signal/noise levels and prevents performing pooled sample analysis with more than eight [19] samples per pool. Moreover, TILLING, like other pre-screening methods does not reveal information about the nature of sequence changes and their possible impact on gene function. Consequently, there is a need for robust and low cost amplicon sequencing methods applicable to large populations.

**Figure 1:**
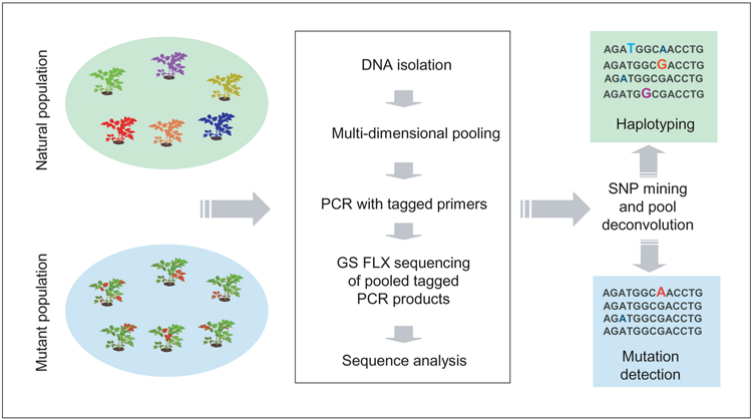
KeyPoint flowchart.

The recent introduction of instruments capable of producing millions of DNA sequence reads in a single experiment opened the possibility of developing a new high-throughput mutation discovery technology based on massive parallel sequencing. Here we describe the KeyPoint™ technology, a novel mutation/polymorphism screening technique using the Genome Sequencer (GS) FLX platform (Roche Applied Science), which allows massive parallel picoliter-scale amplification and pyrosequencing of individual DNA molecules [20]. Using KeyPoint, genes of interest are directly amplified by PCR and sequenced. To significantly reduce sample preparation costs, KeyPoint applies a multidimensional pooling strategy of amplification templates (DNA samples) belonging to mutant or natural populations. Gene-specific PCR primers carry sample identification tags specific for each multidimensional pool in order to assign sequence reads to individual mutant plants or to assign sequence haplotypes to pooled or individual samples of a germplasm collection (Fig. 1). Using custom developed bio-informatics tools the sequence reads are clustered, aligned, and mined for mutations or single nucleotide polymorphisms (SNPs). Statistical probability calculation methods are used to distinguish true mutations and polymorphisms from amplification or sequencing errors. With the KeyPoint technology we identify two EMS induced mutations in exon 1 of the tomato (*Solanum lycopersicum*) eukaryotic translation initiation factor 4E (*SleIF4E*) gene by screening 15,040 mutant tomato plants (five segregating M2 plants of each of 3008 M2 families) in a single GS FLX run. In addition, power calculations were performed to define the throughput of the KeyPoint technology. Finally, KeyPoint revealed at least six naturally occurring haplotypes defined by fifteen SNPs observed in three amplicons of the *SleIF4E* gene in a subset of 92 lines of the EU-SOL tomato germplasm core collection in just 1/4 GS FLX run.

We propose KeyPoint as a generic approach to screen for induced and naturally occurring sequence variation in selected target genes of mutant and/or germplasm populations.

## Materials and Methods

### Plant material

The mutant library used was an isogenic library of inbred tomato cultivar M82 derived from ethyl-methane sulfonate (EMS) mutagenesis treatment. The original characteristics of the library are described by Menda and co-workers [21] and stored in a database on http://zamir.sgn.cornell.edu/mutants/index.html. Seeds of each M2 family were stored at 10% relative humidity at 7°C.

Leaf material was harvested from five individual greenhouse-grown plants of each of 3008 M2 tomato mutant families. As any mutation occurring in the library will segregate in a Mendelian fashion in the M2 offspring, pooling of leaf material of five individual plants reduces the likelihood of not sampling a mutation as consequence of segregation to 0.1% (0.5^10^ = 0.001).

The tomato natural population samples used consists of 92 tomato lines belonging to the core collection of EU-SOL and professor D. Zamir (The Hebrew University of Jerusalem). A description of these 92 lines is provided in figure S1. Characteristics of the tomato core collection can be found on https://www.eu-sol.wur.nl/.

### DNA extraction, normalization and pooling

Genomic DNA was isolated from five pooled leaves obtained from five plants of each M2 family and from leaves of a single plant of each tomato line of the EU-SOL core collection, using a modified CTAB procedure [22]. DNA samples were quantified using Quant-iT™ PicoGreen® dsDNA reagent (Invitrogen) on the FLUOstar Omega (BMG LABTECH) using a standard procedure. DNA samples were diluted to a concentration of 20 ng/nl and subsequently pooled.

For screening the mutant library, M2 DNAs were first subjected to a four-fold pooling, resulting in 752 pooled samples contained in eight 96-well microtiter plates. These 752 four-fold pooled M2 DNA samples were then subjected to a three-dimensional (3D) pooling strategy, such that each sample was represented once in an X, Y and Z-coordinate pool. X pools were assembled by pooling all four-fold pooled M2 DNA samples per column of eight wells (e.g. A1–H1) of the eight 96-well plates and Y pools were assembled by pooling all M2 DNA samples per row of twelve wells (e.g. A1–A12) of the eight 96-well plates. This resulted in 12 X and 8 Y pools which each represented maximum 256 (8×8×4) and 384 (12×8×4) M2 families, respectively. Z pools were assembled by pooling all four-fold pooled DNA samples of an entire 96-well pooled plate, resulting in eight Z pools each representing 384 (4×96) M2 families. For screening 92 tomato core collection lines, genomic DNA samples were subjected to a 2D pooling strategy where X and Y pools were assembled by pooling all DNA samples together per column of eight wells (e.g. A1–H1) or per row of twelve wells (e.g. A1–A12). This resulted in 12 X and 8 Y pools each representing a maximum of eight or twelve tomato lines, respectively.

### KeyPoint amplification targets

The *SleIF4E* gene was chosen as a target sequence for KeyPoint mutation and natural polymorphism detection. Several recessive mutations in this gene are associated with broad resistance to potyviruses in some plant species [23]. At the onset of our research, only the cDNA sequence of *SleIF4E* was known (Genbank AF259801). We employed a polymerase chain reaction (PCR) approach to determine the genomic sequence of *SleIF4E* in part (figure S3), but the genomic sequence has since been deposited in public databases (EMBL CU856538).

Specific primers were designed for PCR amplification of *SleIF4E* exon 1 (amplicon 1; 287 bp), exon 2, intron 2 and exon 3 (amplicon 2; 402 bp), and exon 4, intron 4 and exon 5 (amplicon 3; 200 bp). These primers contained the following target-specific sequences (5′-3′): ATGGCAGCAGCTGAAATGG (amplicon 1 forward primer), CCCCAAAAATTTTCAACAGTG (amplicon 1 reverse primer), TGCTTACAATAATATCCATCAC (amplicon 2 forward primer), CCTGAGCTGTTTCATTTGC (amplicon 2 reverse primer), TTAGCATTGGTAAGCAATGG (amplicon 3 forward primer) and CTATACGGTGTAACGATTC (amplicon 3 reverse primer). Six nucleotide non-target sequences were added at the 5 prime ends of these primers (figure S2). The sample identification tags all differed by at least two nucleotides to exclude the possibility that a single nucleotide substitution error could cause incorrect assignment of the sequence reads to a sample pool [24]. The use of sample identification tags saves sequence library preparation costs and prevents relying on physical compartmentalization of multiple libraries constructed from different sample pools during the sequencing process. Furthermore this approach provides flexibility to process multiple (pooled) samples in one GS FLX run.

### KeyPoint template preparation

*SleIF4E* amplicon 1 was chosen as a target for mutation screening (MS) and *SleIF4E* amplicons 1, 2 and 3 for natural variation screening (NVS). Fifty µl PCR reactions were performed containing 80 ng DNA for each of the 28 3D tomato M2 family pools and each of the 20 2D tomato line pools, 50 ng forward tagged primer, 50 ng reverse tagged primer, 0.2 mM dNTP, 1 U Herculase® II Fusion DNA polymerase (Stratagene) and 1X Herculase® II reaction buffer. PCRs were performed with the following thermal profile: 2 minutes at 95°C, followed by 35 cycles of 30 sec 95°C, 30 sec 56°C and 30 sec 72°C, followed by cooling down to 4°C. Equal amounts of PCR products of the 3D or 2D pooled samples were combined and further treated as one GS FLX fragment library sample.

### GS FLX library preparation and titration

Five and 3.2 µg of the combined PCR fragments (i.e. pooled and tagged PCR products), obtained from mutant- and natural population pools respectively, and were used as input for GS FLX library construction. The use of tagged and pooled PCR products, however, necessitated some adaptations in the published GS library construction protocol [20]. First, no shearing was carried out and second, T4 DNA polymerase treatment, generally used to generate fragment termini suitable for blunt-end ligation, was omitted because PCR fragments were produced using a proof-reading *Taq* polymerase yielding blunt ends. After library construction, emulsion titration and bead enrichment were carried out according to the standard GS FLX protocol (Roche Applied Science).

### GS FLX sequencing

One full picotiterplate (PTP) (70×75 mm) with two regions was used for sequencing the mutant population (EMS) library and 1/4 PTP was used for sequencing of the natural population (EU-SOL) library. Sequencing was performed according to the manufacturer's instructions (Roche Applied Science).

### KeyPoint bio-informatics analysis

**Figure 2:**
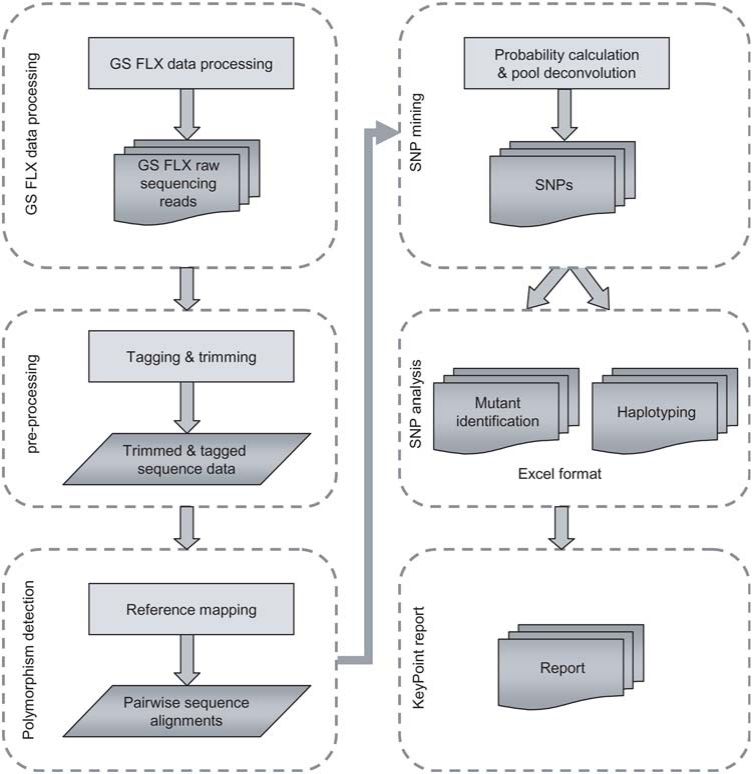
Bioinformatics pipeline for high-throughput analysis of KeyPoint sequence runs.

The mutation/SNP screening process consisted of six parts, namely (1) GS FLX data processing, (2) KeyPoint pre-processing, (3) polymorphism detection, (4) SNP mining, (5) SNP analysis and (6) reporting (Fig. 2).

GS FLX data processing was performed using the Roche GS FLX software (Release: 1.1.03.24). Base-called reads were trimmed and filtered for quality and converted into FASTA format. A total of 666,864 and 95,222 quality filtered reads obtained from the MS and NVS libraries respectively were further processed in the KeyPoint pre-processing step. During pre-processing, the origin of the reads was identified based on the target-specific primer sequences and the six base sample identification tags. This step was implemented in a custom Perl script that used the Semi Global Smith Waterman algorithm within a TimeLogic DeCypher system (Active Motif, Inc.). Furthermore, sample tag and primer sequences were trimmed, and the pre-processed reads of each multidimensional pool were saved separately to the database. In the polymorphism detection step, each pool dataset was mapped against the reference sequence using SSAHASNP [http://www.sanger.ac.uk/Software/analysis/ssahaSNP/] with the -454 and the -NSQ parameters set to true. Raw SSAHASNP output was parsed using a custom Perl script. SNPs derived from the SSAHASNP output were divided in “forward” and “reverse” set depending on the orientation of the read compared to the reference. Both sets were saved in a comma separated format.

For the MS sequence data, the combined set of polymorphisms observed for “forward” and “reverse” orientation reads was filtered on G to A and C to T substitutions only, as these are the changes expected from EMS-induced treatment. On the contrary, for the NVS data analysis all polymorphisms were considered. Using MS-Excel a matrix was built with polymorphism position on the reference sequence as rows and pools as columns. Per position the average polymorphism error rate was calculated by dividing the sum of all polymorphisms over all pools by the total number of reads. Next the probability of finding the observed number of polymorphisms (*k*) was calculated given an *H_0_* of no underlying mutation for each pool at each position. This was done by taking the Poisson distribution with *λ* = *n.p*, *p* being the estimated error rate and *n* being the number of all reads detected per pool. P-values below a significance value of 0.01 indicate pools with true mutants. A combination of two significant 2D or 3 significant 3D coordinate pools, one in each different coordinate, were considered as pointing at (pooled) samples harboring true SNPs in the natural population and true mutations in the mutant population, respectively.

### Detection power / throughput estimation

For one region of the GS FLX run (1/2 picotiterplate) of the mutant population, random subsets of sequence reads were generated with a custom Perl script. Each of the reads was randomly assigned a number between 1 and x for a subset of 1/x of region 1 of the run. Only reads that were assigned a 1 are used in the subset. With this method subsets with a fraction of 1/8, 1/4 and 1/2 of the region 1 MS dataset were generated. Probabilities to detect SNPs in the full MS GS FLX run (1 PTP) and in these subsets (1/2 PTP, 1/4 PTP, 1/8 PTP and 1/16 PTP) were performed as described above, which served as basis for the estimation of KeyPoint detection throughput levels per GS FLX run.

### SNP validation

SNPs were confirmed by Sanger sequencing. Specifically, PCR fragments obtained from the M2 families or the tomato lines carrying mutations or polymorphisms were sequenced using the BigDye®Terminator v.3.1 kit on the 3730xl DNA analyzer (Applied Biosystems) according to standard procedures.

## Results

### EMS mutation screening and validation

**Figure 3:**
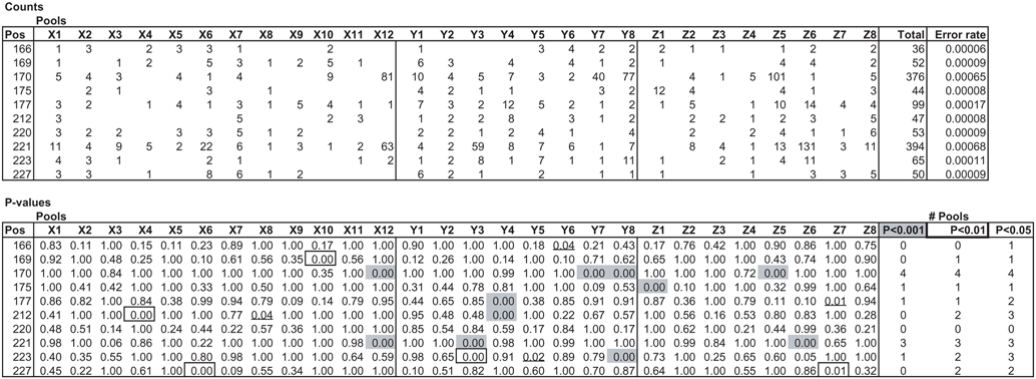
Results KeyPoint analysis mutant population. The top panel shows numbers of G to A (position 221) and C to T (position 170) sequence deviations compared to the wild type sequence observed in each of the 3D pools in a subset of nucleotide positions of the *Sl*eIF4E amplicon 1. The total number of observed sequence deviations and calculated average error rates are shown at the right hand side. The bottom panel shows corresponding P values of false positives for each X, Y and Z pool. Total numbers of pools surpassing significance thresholds P<0.001, P<0.01 and P<0.05 are shown at the bottom right. The complete analysis of all nucleotide positions are shown in figure S4.

**Figure 4:**
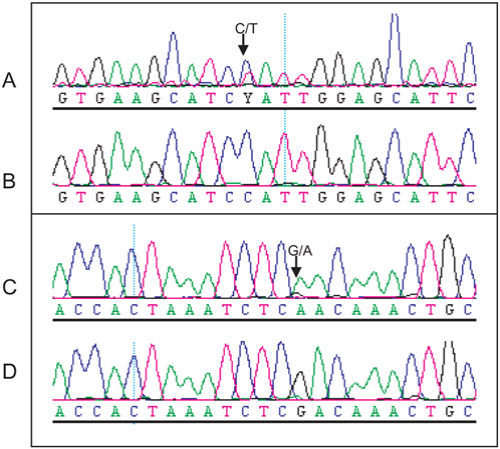
Sanger sequencing confirmation of EMS mutations at positions 170 and 221 of amplicon 1 of the *Sl*eIF4E gene. A) C→T mutation at position 170. B) C at position 170 in wild type sequence. C) G→A mutation at position 221. D) G at position 221 in wild type sequence.

A total of 15,000 M2 plants representing 3008 M2 families were screened for EMS-induced mutations in exon 1 of the *SleIF4E* gene based on amplification from 28 3D pools (12X, 8Y and 8Z). A total of 667,864 high-quality sequence reads with an average read length of 254 bases were obtained from a single GS FLX run. In the pre-processing step (Fig. 2), sample identification tags could be assigned with confidence to a total of 580,471 reads (87%, Table 1). The remaining 13% of reads contained one or more deviations in the sample identification tag sequences, contained concatamers or were reads shorter than 100 bases, and were excluded from further analysis. Successfully trimmed and tagged reads were taken into the mutation/polymorphism mining step starting with mapping them onto the reference sequence and followed by generating pair-wise sequence alignments (Fig. 2). Next, the numbers of C→T and G→A changes from the wild type sequence were counted for each position per pool (Fig. 3) and the probabilities that they represent true EMS mutations were calculated taking into account their distribution across the 3D sample pools. At significance threshold P<0.01, two mutations were identified: a C→T mutation at position 170 and a G→A mutation at position 221, which encode a proline to leucine (both hydrophobic amino acids) and arginine (positively charged and hydrophilic) to glutamine (hydrophilic) amino acids changes, respectively (figure S3). These mutations were based on significantly elevated numbers of non-wild type nucleotides at positions 170 and 221 in four (X12, Y7, Y8 and Z5) and three pools (X12, Y3 and Z6), respectively (Fig. 3). A complete overview of the statistical analysis is provided in figure S4. Sanger sequencing confirmed the C170T mutation in one of the four M2 families located at the plate position specified by the X12, Y7, Z5 3D pool coordinates, and the G221A mutation in one of the four M2 families at the plate position defined by the X12, Y3 and Z6 coordinates (Fig. 4).

**Table 1:** Overview of results of GS FLX KeyPoint runs in tomato.

KeyPoint Mutation Screening	# amplicons	1
	Total # of reads after filtering (GS-FLX raw sequencing reads	667,864
	Reads with sample identification tag assigned	580,471 (87%)
	Faulty reads	87,393 (13%)
	# identified mutants	2
KeyPoint Natural Variation Screening	# amplicons	3
	Total # of reads after filtering (GS-FLX raw sequencing reads	95,222
	Reads with sample identification tag assigned	80,634 (85%)
	Faulty reads	14,588 (15%)
	Reads with sample identification tag assigned for amplicon 1	31,724 (33.3%)
	# haplotypes	4
	Reads with sample identification tag assigned for amplicon 2	11,494 (12.2%)
	# haplotypes	5
	Reads with sample identification tag assigned for amplicon 3	37,415 (39.3%)
	# haplotypes	3

### Natural polymorphism screening and validation

**Figure 5:**
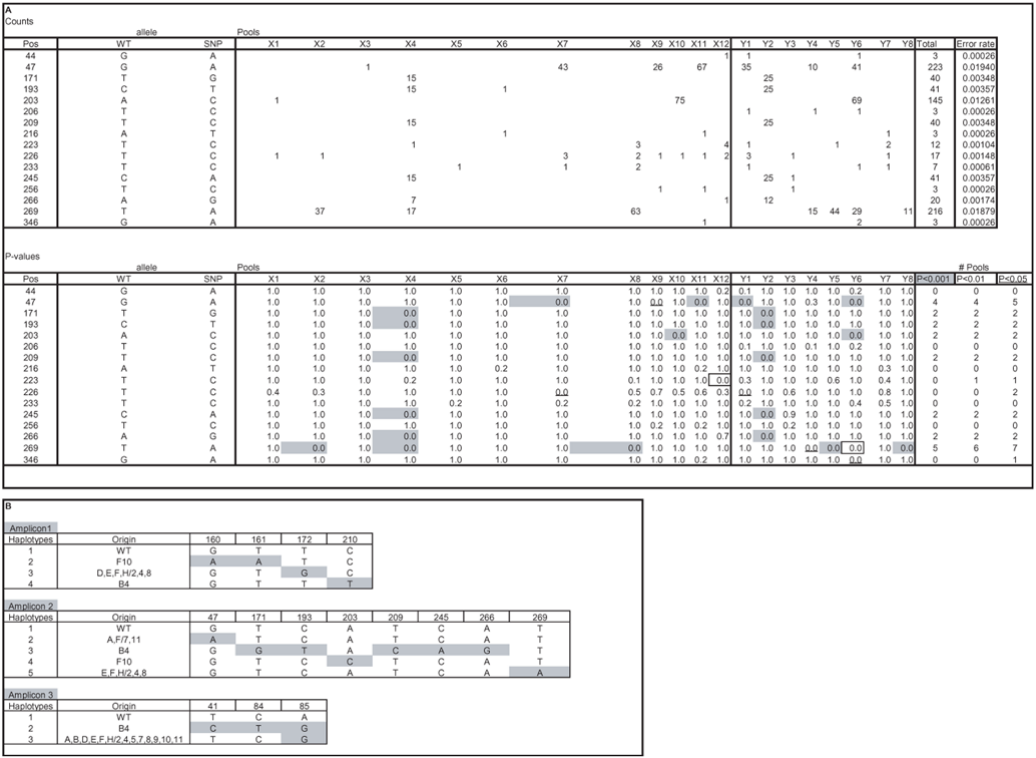
Results KeyPoint analysis tomato natural population EU-SOL core collection. A) Results KeyPoint analysis tomato natural population EU-SOL core collection. The top panel shows numbers of all sequence deviations compared to the wild type sequence observed in each of the 2D pools at a selected subset of nucleotide positions of the *Sl*eIF4E amplicon 2. The total number of observed sequence deviations and calculated average error rates are shown at the right hand side. The bottom panel shows the corresponding P values for each of the X and Y pools. Total numbers of pools surpassing significance thresholds P<0.001, P<0.01 and P<0.05 are shown at the bottom right. The complete analysis of all nucleotide positions of amplicons 1, 2 and 3 is shown in figure S5a, S5b and S5c, respectively. B) SNPs and haplotypes of the *Sl*eIF4E gene observed in 92 lines of the EU-SOL tomato core collection. Haplotype 1 is wild type (WT) sequence. Alleles different from WT are shown in grey. Nucleotide positions in amplicons 1, 2 and 3 are shown at the top. 96-well plate row (A–H) and column (1–12) positions containing samples carrying haplotypes are shown in the “origin” column.

A total of 92 tomato lines of the EU-SOL core collection were screened for natural variation in exons 1–5 and introns 2 and 4, based on PCR amplification of three amplicons of the *SleIF4E* gene (Figure S3) from 20 (12X and 8Y) 2D pools using tagged PCR primers. Amplicon sizes were as expected 287 bp, 402 bp and 200 bp, respectively, excluding non-template tags. A total of 95,222 high-quality sequence reads with an average read length of 230 bases were obtained from a 1/4 region of a GS FLX PTP. Sample identification tags could be assigned with confidence to a subset of 80,634 of these reads (85%, Table 1) as described above for the EMS mutation screening. This included 31,724 amplicon 1 reads, 11,495 amplicon 2 reads and 37,415 amplicon 3 reads (Table 1). All nucleotide changes from the *SleIF4E* wild type sequence were counted for each of 20 2D pools for all positions in all three amplicons (figure S5). A subset of the data obtained from amplicon 2 is shown in figure 5a, which includes all eight positions (47, 171, 193, 203, 209, 245, 266 and 269) where SNPs were found, and a number of non-polymorphic positions for comparison purposes. As expected for a 2D pool design, statistically significant probabilities for harboring true mutations (P<0.01) were observed in at least 2 pools (one X and one Y) for each of these eight SNPs (Fig. 5a). Similar analyses performed for amplicons 1 and 3 revealed four and three SNPs, respectively (figure S5). All 15 SNPs observed in amplicons 1–3 together were confirmed by Sanger sequencing of selected PCR products of individual samples (*data not shown*).

The combinations of SNPs in amplicons 1, 2 and 3 defined four, five and three sequence haplotypes, including the wild type haplotype (Fig. 5b). Excluding the wild type haplotype, two amplicon 1 haplotypes, two amplicon 2 haplotypes and one amplicon 3 haplotype could be assigned to individual 2D pooled samples (i.e. tomato lines) in the 96-well base plate, at positions B4 and F10 for amplicons 1 and 2, and position B4 for amplicon 3, respectively (Fig. 5b). Taken together, the three amplicons therefore defined at least six *SleIF4E* haplotypes in the collection of 92 tomato lines: the wildtype haplotype ( = amplicon 1 haplotype 1 – amplicon 2 haplotype 1 – amplicon 3 haplotype 1; Figure 5b), and similarly for amplicons 1, 2 and 3 the haplotype number combinations, 1-2-(1 or 3), 2-4-(1 or 3) (sample F10), 3-1-(1 or 3), 3-5-(1 or 3) and 4-3-2 (sample B4) as shown in Figure 5b.

### Detection power / throughput estimation

**Figure 6:**
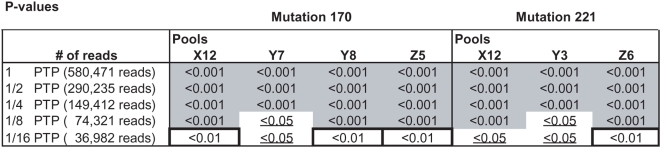
P-values of detecting mutations C170T and G221A in progressively smaller subsets of the total of 580,471 sequence reads obtained from a single GS FLX run.

The total number of 580,471 trimmed and tagged sequences reads of the EMS mutation screening of *SleIF4E* exon 1 was used to determine the power of detection of the two identified mutations at positions 170 and 221 in progressively smaller subsets of the total dataset. For this, random subsets of the data were generated representing 1/2, 1/4, 1/8 and 1/16 fractions of all reads (Fig. 6). These subsets were subjected to statistical analysis and probability calculations to identify mutations as described above for the whole dataset. The results shown in Figure 6 indicate that both EMS mutations can be found with the same statistical confidence levels (P<0.001) in all positive pools with just a 1/4 fraction of all sequence data. In the positive X and Z pools harboring mutations C170T and G221A, this is even the case with a 1/8 fraction of the data, but for the Y pools, significance levels drop to P<0.05, which is below the cut-off level P<0.01 which we considered reliable to detect true mutations.

Given the fact that we screened one amplicon in 3008 M2 families in one GS FLX run, the estimated throughput of KeyPoint screening equals four amplicons screened in 3008 M2 families per GS FLX run, assuming that these amplicons are represented in equal proportions, or four times as many M2 families for a single amplicon.

## Discussion

We have applied KeyPoint technology to identify mutations in the *SleIF4E* gene in an EMS population of 3008 M2 families and SNPs in a selection of 92 tomato lines of the EU-SOL core collection, taking advantage of the power of the GS FLX next generation sequencing platform. The EMS mutation screening of *SleIF4E* exon 1 involved only 28 PCRs with tagged primers on 3D pooled samples, and led to the discovery of two transition mutations which were confirmed by Sanger sequencing. Both mutations are predicted to confer amino acid changes but their impact on gene function is unknown.

During the development of the KeyPoint technology, we noticed that the number of sequence changes compared to the wild type sequence, which reflects the combined total of PCR- and sequence errors and genuine EMS mutations, was highly variable across all positions of the amplicon sequence. Moreover, these numbers were also rather variable between “forward” and “reverse” orientation GS FLX reads of the amplicon (*data not shown*). Based on this observation, which has been made previously by others [25], [26], [27], we concluded that identification of EMS mutations or SNPs could not be performed reliably based on the total number of sequence deviations from the wild type sequence alone, without considering their distribution across the individual multidimensional pools. Consequently, a probability calculation method based on Poisson statistics was established which takes this distribution into account. Using this method, both EMS mutations and fifteen SNPs identified in the natural population screening were confirmed when a threshold significance level of P<0.01 was applied.

Unexpectedly for a 3D pooling scheme, for the position C170T EMS mutation in *SleIF4E* exon 1, four positive pools were observed (X12, Y7, Y8 and Z5). This includes two Y-dimension coordinates and specifies two unique plate addresses: X12, Y7, Z5 and X12, Y8, Z5. In fact the mutation was confirmed in one of four M2 families of the first address, whereas the second one pointed to an empty (adjacent) well, despite the fact that 77 C→T changes were observed in Y8 compared to 40 in Y7 (Fig. 3). We can only explain this observation by an experimental error made either during (manual) assembly of the 3D pools or setting up the PCRs. Despite this flaw, the C170T mutation was identified and assigned to the correct position.

Based on analysis of progressively smaller subsets of the EMS data, we estimated that KeyPoint technology enables screening of four amplicons in 3000 M2 families per GS FLX run of approximately 500,000 sequence reads. The maximum read length of these amplicons is defined by the specifications of GS FLX platform (approximately 240 bases). Hence, a total of 4×3000×240 bases equalling 2,88 Mb of amplicon sequence can be screened in a single GS FLX run. Obviously, other combinations of sample- and amplicon numbers within these boundaries can be considered as well, with appropriate adaptation of the pooling strategy. The throughput will increase with further output improvements of the GS FLX platform. For example, the recently released GS FLX *Titanium* platform yields around 1 million sequence reads of >400 bases, which increases the throughput of KeyPoint screening to (the equivalent of) 4×6000 M2 plants×400 bases equating 9.6 Mb of screened amplicon sequence per GS FLX *Titanium* run.

Based on these results, we conclude that KeyPoint screening offers a sequence-based alternative to TILLING (and EcoTILLING) screening which comes with certain advantages: 1) the sequence context of mutations is determined which is important when screening for knock-out mutations, especially given the fact that only a minor fraction of EMS mutations is expected to confer a stop codon or splice site errors [8]. 2) KeyPoint is robust and does not rely on endonucleases with known robustness issues. 3) KeyPoint does not rely on visual inspection or interpretation of slab gel data aided by image analysis software, but is based on an objective statistical analysis method which is easy to perform. 4) The method is flexible with respect to changing numbers of samples and amplicons. 5) Compared to unidirectional Sanger sequencing, KeyPoint is based on highly redundant sequencing of amplicons, which improves the accuracy when appropriate analysis methods are applied; this in turn reduces the likelihood of identifying false positives due to imperfections of Sanger sequencing. 6) Costs are scalable as it is not mandatory required to perform an entire GS FLX (*Titanium*) run, but picotiterplate containing multiple compartments are available for use as well.

We also applied KeyPoint to investigate germplasm diversity in a small subset of the tomato EU-SOL core collection as a pilot project. A total of fifteen SNPs and at least six sequence haplotypes were found based on joint analysis of three amplicons of the *SleIF4E* gene and applying a 2D pooling scheme. The impact on these haplotypes on gene function is unknown. As for the EMS screening, this approach can be scaled up for analysis of larger numbers of samples, with limited additional efforts and costs for amplicon preparation. Although germplasm diversity can also be revealed based on sequencing of a single pooled sample, an advantage of using a 2D (or higher order) pooling scheme, is that a subset of identified SNPs and haplotypes can be attributed to a specific sample, or at least a subset of rows and columns / pool coordinates, while sample preparation costs are only marginally higher. This is not the case when all samples are pooled together. In fact, for rare SNPs and haplotypes in the germplasm which are often the most interesting to discover, there is a higher probability to identify the associated sample than for more common polymorphisms, as shown by our results. A second advantage of using a multidimensional pooling strategy is that it provides a built-in quality control capable of separating genuine polymorphisms from experimental (PCR and sequencing) errors, since true mutations must be observed in at least two dimensions in case of a 2D design. Also this is not the case when all samples are pooled together. These features of KeyPoint technology for screening natural variation compare favourably to a number of alternative (pre-screening) technologies, which may lack the power to detect low frequency polymorphisms [6], [7].

In conclusion, we present KeyPoint technology as a flexible, high-throughput sequence-based polymorphism screening technology, applicable for detection of artificially induced and natural polymorphisms in a wide variety of species.

## Supporting Information

Figure S1 Tomato lines of the EU-SOL core collection used for screening naturally occurring variants in the *SleIF4E* gene.
(0.01 MB PDF)

Figure S2 Sample identification tags.
(0.00 MB PDF)

Figure S3 *SleIF4E* sequence, including positions of discovered mutant and naturally occuring SNPs.
(0.01 MB PDF)

Figure S4 Results KeyPoint analysis mutant population.
(0.02 MB PDF)

Figure S5 Results KeyPoint analysis on natural populations.
(0.03 MB PDF)

## References

[1] Ahloowalia BS, Maluszynsky M, Nichterlein K (2004). Global impact of mutation-derived varieties.. Euphytica.

[2] Hestekin CN, Barron AE (2006). The potential of electrophoretic mobility shift assays for clinical mutation detection.. Electrophoresis.

[3] Montgomery J, Wittwer CT, Palais R, Zhou L (2007). Simultaneous mutation scanning and genotyping by high-resolution DNA melting analysis.. Nat Protocols.

[4] Den Dunnen JT, van Ommen GJ (1999). The protein truncation test: A review.. Hum Mutation.

[5] Taylor GR (1999). Enzymatic and chemical cleavage methods.. Electrophoresis.

[6] Jenkins GJS (2004). The restriction site mutation (RSM) method: clinical applications.. Mutagenesis.

[7] Li J, Berbeco R, Distel RJ, Jänne PA, Wang L (2007). s-RT-MELT for rapid mutation scanning using enzymatic selection and real time DNA-melting: new potential for multiplex genetic analysis.. Nucleic Acids Res.

[8] McCallum CM, Comai L, Greene EA, Henikoff S (2000). Targeted screening for induced mutations.. Nat Biotechnol.

[9] Colbert T, Till BJ, Tompa R, Reynolds S, Steine MN (2001). High-throughput screening for induced point mutations.. Plant Physiol.

[10] Gilchrist EJ, Haughn GW (2005). Tilling without a plough; a new method with application for reverse genetics.. Curr Opinion Plant Biol.

[11] Comai L, Young K, Till BJ, Reynolds SH, Greene EA (2004). Efficient discovery of DNA polymorphism in natural populations by Ecotilling.. Plant J.

[12] Sood R, English MA, Jones M, Mullikin J, Wang DM (2006). Methods for reverse genetic screening in zebrafish by resequencing and TILLING.. Methods.

[13] Gilchrist E, O'Neil N, Rose A, Zetka M, Haughn G (2006). TILLING is an effective reverse genetics technique for Caenorhabditis elegans.. BMC Genomics.

[14] Greene EA, Codomo CA, Taylor NE, Henikoff JG, Till BJ (2003). Spectrum of chemically induced mutations from a large-scale reverse-genetic screen in Arabidopsis.. Genetics.

[15] Suzuki T, Eiguchi M, Kumamaru T, Satoh H, Matsusaka H (2008). MNU-induced mutant pools and high performance TILLING enable finding of any gene mutation in rice.. Mol Genet Genomics.

[16] Cooper JL, Till BJ, Laport RG, Darlow MC, Kleffner JM (2008). TILLING to detect induced mutations in soybean.. BMC Plant Biology.

[17] Slade AJ, Fuerstenberg SI, Loeffler D, Steine MN, Facciotti D (2005). A reverse genetic, non-transgenic approach to wheat crop improvement by TILLING.. Nat Biotechnology.

[18] Till BJ, Reynolds SH, Weil C, Springer N, Burtner C (2004). Discovery of induced point mutations in maize genes by TILLING.. BMC Plant Biology.

[19] Till B J, Zerr T, Comai L, Henikoff S (2006). A protocol for TILLING and Ecotilling in plants and animals.. Nature Protocols.

[20] Margulies M, Egholm M, Altman WE, Attiya S, Bader JS (2005). Genome sequencing in microfabricated high-density picolitre reactors.. Nature.

[21] Menda N, Semel Y, Peled D, Eshed Y, Zamir D (2004). In silico screening of a saturated mutation library of tomato.. Plant J.

[22] Stuart CN, Via LE (1993). A rapid CTAB DNA isolation technique useful for RAPD fingerprinting and other PCR applications.. Biotechniques.

[23] Ruffel S, Gallois JL, Lesage ML, Caranta C (2005). The recessive potyvirus resistance gene pot-1 is the tomato orthologue of the pepper pvr2-eIF4E gene.. Mol Gen Genomics.

[24] van Orsouw NJ, Hogers RC, Janssen A, Yalcin F, Snoeijers S (2007). Complexity Reduction of Polymorphic Sequences (CRoPS™): A Novel Approach for Large-Scale Polymorphism Discovery in Complex Genomes.. PLoS One.

[25] Huse SM, Huber JA, Morrison HG, Sogin ML, Welch DM (2007). Accuracy and quality of massively parallel DNA pyrosequencing.. Genome Biology.

[26] Wang C, Mitsuya Y, Gharizadeh B, Ronaghi M, Shafer RW (2007). Characterization of mutation spectra with ultra-deep pyrosequencing: application to HIV-1 drug resistance.. Genome Res.

[27] Hoffmann C, Minkah N, Leipzig J, Wang G, Arens MQ (2007). DNA bar coding and pyrosequencing to identify rare HIV drug resistance mutations.. Nucleic Acids Res.

