# Supplementary material for: High-Throughput Detection of Induced Mutations and Natural Variation Using KeyPoint™ Technology
Source: PLoS One. 2009 Mar 13;4(3):e4761. doi: 10.1371/journal.pone.0004761 (PMC2654077; doi:10.1371/journal.pone.0004761)
Supplement: Figure S1 — Tomato lines of the EU-SOL core collection used for screening naturally occurring variants in the SleIF4E gene. (0.01 MB PDF) [file pone.0004761.s001.pdf]

microtiter position	Variety name	EU-SOL number	Origin
A1	LIME GREEN SALAD	EA00971	Unknown
C1	AVIURI	EA01106	COSTA RICA
D1	NOTA	EA01228	HONDURAS
F1	BRANSCOMB'S ORANGE	EA01336	MEXICO
G1	Egizia	EA02048	ITALY
H1	Lycopersicum esculentum Mill.fruticosum Lehm.finiens Lehm	EA02124	GUATEMALA
A2	Ottawa T.3 Lycopersicon esculentum Mill.	EA02726	CANADA
C2	Lycopersicum esculentum Mill.fruticosum Lehm.finiens Lehm	EA02769	COSTA RICA
D2	Lycopersicum esculentum Mill.	EA02860	EL SALVADOR
F2	Lycopersicum esculentum Mill.	EA02923	EL SALVADOR
G2	Plan 904	EA03136	Unknown
H2	Progress	EA03148	Unknown
A3	Ladoga	EA03176	Unknown
B3	M82	EA06884	Unknown
C3	Roma (1445) V.F.	EA03366	UNITED STATES
D3	Titan	EA01198	HONDURAS
E3	Ildi	EA00361	Unknown
F3	Renate	EA03548	GERMANY
G3		EA03652	Italy
H3	Walter	EA03667	Unknown
A4		EA03686	Italy
B4	S.pennellii (LA716)	EA10008	Unknown
C4	21162	EA05659	COLOMBIA
D4	Heinz 1630 VF	EA05783	ITALY
E4	W-C 1579	EA06150	GUATEMALA
F4	EU-DR 14	EA06647	Unknown
G4	anna herman	EA00010	Unknown
H4	ashleigh	EA00012	Unknown
A5	black cherry	EA00027	Unknown
B5	s.PIMPINELLIFOLIUM (LA1589)	EA01467	Spain
C5	ESSEX WONDER	EA00056	Unknown
D5	GEORGIA PEACH	EA00064	Unknown
E5	HUMPH	EA00084	Unknown
F5	NOIR CHARBONNEUSE	EA00135	Unknown
G5	VERA'S SEEDS OF PARADISE	EA00208	Unknown
H5	YASHA	EA00218	Unknown
A6	YELLOW RIESENTRAUBE	EA00223	Unknown
B6	ANANAS NOIRE	EA00258	Unknown
C6	BLACK TOM	EA00281	Unknown
D6	BLONDOKEE	EA00282	Unknown
E6	COPIA	EA00299	Unknown
F6	COYOTE	EA00302	Unknown
G6	DOROTHY'S GREEN	EA00312	Unknown
H6	ISBELL'S GOLDEN COLOSSAL	EA00365	Unknown
A7	KATINKA CHERRY	EA00375	Unknown
B7	MALSCHOR ISURA	EA00395	Unknown
C7	MAYO'S DELIGHT, FLEMMO	EA00402	Unknown
D7	POTATO LEAF TYPE	EA00449	Unknown
E7	ROGER'S BEST BLACK	EA00459	Unknown
F7	SAUERLING	EA00466	Unknown
G7	STUMP OF THE WORLD	EA00480	Unknown
H7	VINTAGE WINE	EA00506	Unknown
A8	L.esculentum Tehuacan, Puebla, Mexico	EA00658	MEXICO
B8	MARISE	EA00815	Unknown
C8	MARMANDE HATIVE	EA00820	Unknown
D8	Panadaro (IND)	EA00881	GUATEMALA
E8	Sponzillo (IND)	EA00882	GUATEMALA
F8	MIRABELL	EA00933	BRAZIL
G8	RIESENTRAUBE	EA00944	BRAZIL
H8	GREEN ZEBRA	EA00970	BRAZIL
A9	MOLDOVAN GREEN	EA00972	BRAZIL
B9	RUBY TREASURE	EA00973	BRAZIL
C9	OLENA UKRANIAN	EA01063	Unknown
D9	ANGORA	EA01104	COSTA RICA
E9	BIG BITE	EA01113	COSTA RICA
F9	GERMAN GOLD	EA01344	CANADA
G9	KELLOGG'S BREAKFAST	EA01355	BRAZIL
H9	CERASIFORME	EA01500	SPAIN
A10	WATERMELON BEEFSTEAK	EA01640	UNITED STATES
B10	Lycopersicom esculentum Mill.	EA01752	Unknown
C10	Lycopersicom esculentum Mill.	EA01828	Unknown
D10	Alcobaca	EA01926	UNITED STATES
E10	Local	EA01964	SPAIN
F10	PI 129154	EA01966	ECUADOR
G10	Opolchenetc	EA01979	Unknown
H10	Mao Tao Shi Zi	EA02021	Unknown
A11	Palazai tf	EA02033	HUNGARY
B11	Cordiforme	EA02045	Unknown
C11	Lycopersicon esculentum Mill.scopigerum Lehm	EA02064	GUATEMALA
D11	Lycopersicon esculentum Mill.infiniens Lehm	EA02070	GUATEMALA
E11	Lycopersicon esculentum Mill.infiniens Lehm	EA02075	GUATEMALA
F11	Lycopersicon esculentim Mill.scopigerum Lehm	EA02082	GUATEMALA
G11	Dwarf Red Lycopersicon esculentum Mill. Infiniens	EA02116	UNITED STATES
H11	Magnif Olympica Lycopersicon esculentum Mill.	EA02162	ARGENTINA
A12	Tempo Lycopersicon esculentum Mill.infiniens	EA02198	POLAND
B12	Battipaglia 0757 Lycopersicon esculentum Mill.	EA02216	Unknown
C12	Purpurk_nig Lycopersicon esculentum Mill.	EA02284	Unknown
D12	Tezier Prim Lycopersicon esculentum Mill.	EA02295	Unknown
E12	Lycopersicon esculentum Mill.	EA02311	RUSSIAN FEDERATION
F12	Sweet 100 Lycopersicon esculentum Mill.	EA02331	Unknown
G12	Valenciano Lycopersicon esculentum Mill.sco…	EA02550	Unknown
H12	Sulac Lycopersicon esculentum Mill.	EA02556	THE NETHERLANDS
