# Supplementary material for: High-Throughput Detection of Induced Mutations and Natural Variation Using KeyPoint™ Technology
Source: PLoS One. 2009 Mar 13;4(3):e4761. doi: 10.1371/journal.pone.0004761 (PMC2654077; doi:10.1371/journal.pone.0004761)
Supplement: Figure S2 — Sample identification tags. (0.00 MB PDF) [file pone.0004761.s002.pdf]

Sample identification tags (5'-3')	Pool
GATCGT	X1
GAGTCT	X2
GAGATG	X3
GAGCAT	X4
GAGTAG	X5
GATACT	X6
GATCAG	X7
GATGTG	X8
GCACGT	X9
GCACTG	X10
GCAGAT	X11
GCAGCG	X12
GCATAG	Y1
GCGACT	Y2
GCGCAG	Y3
GCGTAT	Y4
GCGTCG	Y5
GCTAGT	Y6
GCTATG	Y7
GCTCAT	Y8
GCTGAG	Z1
GTACAT	Z2
GTAGAG	Z3
GTATCG	Z4
GTATGT	Z5
GTCACT	Z6
GTCATG	Z7
GTCTAT	Z8
