# Supplementary material for: High-Throughput Detection of Induced Mutations and Natural Variation Using KeyPoint™ Technology
Source: PLoS One. 2009 Mar 13;4(3):e4761. doi: 10.1371/journal.pone.0004761 (PMC2654077; doi:10.1371/journal.pone.0004761)
Supplement: Figure S3 — SleIF4E sequence, including positions of discovered mutant and naturally occuring SNPs. (0.01 MB PDF) [file pone.0004761.s003.pdf]

```
  1 atggcagcagctgaaatggagagaacgatgtcgtttgatgcagct   Exon 1
    M  A  A  A  E  M  E  R  T  M  S  F  D  A  A
 46 gagaagttgaaggccgccgatggaggaggaggagaggtagacgat
    E  K  L  K  A  A  D  G  G  G  G  E  V  D  D
 91 gaacttgaagaaggtgaaattgttgaagaatcaaatgatacggca
    E  L  E  E  G  E  I  V  E  E  S  N  D  T  A
                                160-1       170 172
136 tcgtatttagggaaagaaatcacagtgaagcatccattggagcat
                                aa          t  g
    S  Y  L  G  K  E  I  T  V  K  H  P  L  E  H
                            K           L  V
                                   210         221
181 tcatggacttttggtttgataaccctaccactaaatctcgacaa
                                   t           a
    S  W  T  F  W  F  D  N  P  T  T  K  S  R  Q
                               T           Q
226 actgcttggggaagctcacttcgaaatgtctacactttctccact
    T  A  W  G  S  S  L  R  N  V  Y  T  F  S  T
271 gttgaaaattttggggt
    V  E  N  F  W  G

                        gcttacaataatatccatcacccaagc   Exon 2
                        A  Y  N  N  I  H  H  P  S
                        47
316 aagttaattatgggagcagactttcattgttttaagcacaaaatt
                        a
    K  L  I  M  G  A  D  F  H  C  F  K  H  K  I
                      N
361 gagccaaagtgggaagatcctgtatgtgccaatggagggacgtgg
    E  P  K  W  E  D  P  V  C  A  N  G  G  T  W
406 aaaatgagttttcgaagggtaaatctgataccagctggctgtat
    K  M  S  F  S  K  G  K  S  D  T  S  W  L  Y
             171                          193        203
451 acggtattccgaagttatttccatccagcccttaatgataggtca   Intron 2
         g                            t          c
    T
209                                            245
    ttctagtaatgttattttcccctttgatataatttccactcttgt
    c                                          a
                266 269
    tttcttatatggaattattgtagctgctggcaatgattggacat    Exon 3
                g   a
                                 L  L  A  M  I  G  H
    caattcgatcatggagatgaaatttgtggagcagttgttagtgtc
    Q  F  D  H  G  D  E  I  C  G  A  V  V  S  V
496 cgggctaagggagaaaaaatagctttgtggaccaagaatgctgca
    R  A  K  G  E  K  I  A  L  W  T  K  N  A  A
541 aatgaaacagctcag
    N  E  T  A  Q
                                             gttagc   Exon 4
                                             V  S
                                          41
586 attggtaagcaatggaagcagtttctagattacagtgattcggtt
                                          c
    I  G  K  Q  W  K  Q  F  L  D  Y  S  D  S  V
                                       S
```

```
                                                    84 85
631 ggcttcatatttcacgtatgaaatcttggttatcatacgcctttta  Intron 4
                                     tg
    G  F  I  F  H
    attcagtttctcttcaattagcaagactcataaagaatcatcttc
    ttttgcaggacgatgcaaagaggctcgacagaaatgccaagaat    Exon 5
            D  D  A  K  R  L  D  R  N  A  K  N

676 cgttacaccgtatag 696
    R  Y  T  V  *
```
