# Supplementary material for: High-Throughput Detection of Induced Mutations and Natural Variation Using KeyPoint™ Technology
Source: PLoS One. 2009 Mar 13;4(3):e4761. doi: 10.1371/journal.pone.0004761 (PMC2654077; doi:10.1371/journal.pone.0004761)
Supplement: Figure S4 — Results KeyPoint analysis mutant population. (0.02 MB PDF) [file pone.0004761.s004.pdf]

KeyPoint MS analysis - all data combined

Observed counts per position per pool

Sums per pool

	28521	18590	7200	10845	18399	26835	35016	9262	13897	11751	9045	17606	36802	19768	19970	21854	23648	19808	13802	23442	11846	23038	8665	9272	37386	55945	4923	43335		
pos	X1	X2	X3	X4	X5	X6	X7	X8	X9	X10	X11	X12	Y1	Y2	Y3	Y4	Y5	Y6	Y7	Y8	Z1	Z2	Z3	Z4	Z5	Z6	Z7	Z8	Total	error rate
26	3	1			1	1	1	1	1		1		4	2	3	1		1		3	1	1		3	1	4	1	10	45	0.00008
27					1								2			2				1								1	7	0.00001
30					1	1	4																					1	7	0.00001
32	2	3	1	1	2	4	4		1						3	3	3	2				1	1		5	4		2	42	0.00007
33	1					1						1	2				1	1							2			2	11	0.00002
37	1	1	1		3		3	3		10		4	6	1		2	2	3			1	1			1	2		3	48	0.00008
40	3	21				7	5		1	1			2		3	2	1	4	4	3	1	2		2	5	10	1	1	79	0.00014
41	67	65	13	31	33	61	61	22	29	30	29	54	82	34	37	31	32	51	34	65	21	47	21	29	84	134	19	95	1311	0.00226
43	17	5	4	2	5	11	19	2	10	5	2	6	16	10	5	10	7	7		15	5	8	4	2	10	34	2	23	246	0.00042
44	5	1		2	3	4	2	1		1		3	2	1		1		4	2	3	1	1		1	6	11	1	10	66	0.00011
46	1	1		1	1	1	4	3		1	2	2	4	2	3	2	3	5		4		1	1		4	4		1	51	0.00009
48	3	1	2	1	3	2	2	2	1		2	3	1	6	3	1	2	2	2	1	1	3	1		3	11	1	4	64	0.00011
51	5	3	1	1	2	2	11	3	1	3	2	5	8	1	6	3	5	2	2	5	2	3		1	4	5	1	3	90	0.00016
54	2	5		1	1	3	7			1	2	1	5		1		4	1	2	1		5		2	5	1	2	14	66	0.00011
57	5	1	1	3		8	8	5	3	1			3	4	5	1	2	4		3	3	3		5		6		5	79	0.00014
58	15	1			2	3	6		2	3			2	2	3	2	1	1	1		4		3	2	1	5		9	68	0.00012
59	37	46	7	10	10	26	36	6	13	8	8	17	34	34	11	14	22	20	5	22	11	28	3	18	37	63	4	49	599	0.00103
60	2	6	3	1		5	6	2	3			4	1	2	1	1	4	1	2	7	3	3		1	4	11	2	8	83	0.00014
61	11	10	5	2	11	11	14	1	11	6	3	9	13	11	14	10	13	6	8	13	2	10	1	5	16	25		25	266	0.00046
62	8	11	5	5	11	6	15	4	3	10	6	7	14	8	7	13	13	11	6	13	2	5	2	8	14	26	1	17	251	0.00043
63	1	4			3	7	3	1	1	3		4	5	2	7	1		3	1	3	2	1		1	4	1	1		59	0.00010
64	4	5	2	1	1	3	6	3	1		1	2	6	3		3	3	1	5	5	5	4	1		8	3		10	86	0.00015
67	4				2	1	2	3	1	3		2		1	2	2	1	4	4	4		1	1	2	5	9	1	6	61	0.00011
68	7	5	4	2	3	8	11	7	1	8	5	12	7	5	18	6	4	2	12	8	10	3		1	6	7	1	4	167	0.00029
70	4	2			3	1	1	1	1	2			7	1		3	2	1	3	1	2	4	1	1	4	8		4	57	0.00010
71	4	6	2	5	10	5	9	7		8		6	6	1	12	3	10	4	9	8	9	3			2	7		5	141	0.00024
73		1		3		4	4	2		1	2	3	7	5		4	1	1	3	1	1	3			5	4		3	58	0.00010
74		1		1	5	3	2	2					2	1		3	4	5	1	5		2	1	1	4	8	1	7	59	0.00010
76	2	1			1	1	1			3		3	2	4	3	2		1	1	4	3	5			2	6		1	46	0.00008
77	2	3			2	2	3		4	4		1	2	5	1	4	6	2	6	1	1	2		1	3	3		4	62	0.00011
79	1				1	3	4		3	1	2	3			1	1		3		2	2			1	5	3		2	38	0.00007
81	5		2	2	1	2		2	1	1			4			2	3		1	4	1	6		1	9	8		2	57	0.00010
82	4	2			3	2	4		2			4	4	1			2	5	8		1		7	1	2	2		1	55	0.00009
85	5	2		1		1	2	2					4	1			2	1	4	2	1	2	1	4	7	1	1	2	46	0.00008
87	2	1			1		6			1			6	4	1		3		1	6	1	2			7	4		2	48	0.00008
88	2	4	1	1		1	2	2	1	7		1	12	6	3		4	5	1	1	1		1	2		4	1	4	67	0.00012
91	4	2				1	3		1			3	3	3	1	1	2		2	3	1				3	4	2	1	40	0.00007
94	4	1	1			3	10						2	1		2	2		4		2	2			6	4		6	50	0.00009
97		2		1	1		1			1	1	2	3	1	3	3	1	4		2	2	1			2	2		3	36	0.00006
100	2	4	1				5		1	2	1		5	2	1	4	6	1		2	3	2		2	1	6		3	54	0.00009
103	2	2		3	2	2	3	2		2		3	6	1	3		2	1	3	1	2	4		1	4	3		3	55	0.00009
104	2	2		1	2	1	2	4	3	2	1		4	2	2	2	2	1		2	2	1	1	1	3	2		3	48	0.00008
106	3	2	2		2	2	2					1		2	1	2	1	1				1			1	2		2	27	0.00005
112	2	5	1		2	1	2		2			8	4	4	4			2			1	3		3	1	1		1	47	0.00008
115	1	5	1			1	4					3	4	2	1	1	4	1		9	1	1			3	4		1	47	0.00008
118		1	2	1	1	1	2		1	2		3	1	2	5	2	3	2		1	1				2	4		2	39	0.00007
122		1		2		3	1						1	1	2	4	2	1	2	1		1			4	4	1	1	32	0.00006
127		1					5	3		1		1		1	1	2		1	1		1	1		2	1	1		1	24	0.00004
131	14	7		1	1	15	4	4	3	1	3	5	2	6	7	7	3	6	4	2	3	5	5	5	11	17		11	152	0.00026
132	1	5		1	3	3	4	1	1	2		1	1	5	4	1	2	3	1	1	2	2	2	1	7	3		5	62	0.00011
133	158	93	41	37	89	130	155	35	53	50	41	76	190	100	84	65	108	85	55	95	76	110	36	51	189	287	29	209	2727	0.00470
134	1		2			2	3	2		1			5	4	2	1	8	1	1	1					5	8		4	51	0.00009
137	4	4					1	1	1		2	4	2	1		3	4	1	1	4			1		11	6		8	59	0.00010
138	5		1	2	4	4	9	2	2	7			6	3	2	2	2	3	2	3	2	3	1		6	5		9	85	0.00015
145	1	4		1	1		1		3	4			3	4	4	2	4	1		2	1	5			3	8			52	0.00009
146						5	7	1	1				5	6	1	2	1	2	2	1	2					10		3	49	0.00008
147	2	2	1		2	2	1	2	2	4	4	2	5	2	2		1	1	3	2		1	2		5	1		4	53	0.00009
151	1				2	4	1	2	2			1	3		1	2		1		1	2	1	1		2	1	1	3	32	0.00006
156	9	1	1		1	6			1		2		9		3	1	1	1		1	1	1			4	2	3	3	51	0.00009
158		7			3	5	7	1	2		3	2	5	20	3	2	2	2		4	1	1	2	3	5	10	1	6	97	0.00017
160	5	2	2	2	3	5	2	1			2	1	2	1	1	1	2	1	2	4	2	1		1	4	7	1	5	60	0.00010
162	3	1			1	2		1	1	1			1	2	2	1	4	3		2				1	2	12		4	44	0.00008
165	5	1	2	1	2	1	5	1	1	3		5	4	2	6	3	1			6	2	5	1	1	5	15	1	2	81	0.00014
166	1	3		2	3	3	1			2			1				3	4	2	2	2	1	1		1	2		2	36	0.00006
169	1		1	2		5	3	1	2	5	1		6	3		4		4	1	2	1				4	4		2	52	0.00009
170	5	4	3		4	1	4			9		81	10	4	5	7	3	2	40	77		4	1	5	101	1		5	376	0.00065
174	2	7		3	1	3	4	2	1	3		3	3	2	3	2	2	1	1	5			2		3	5		3	61	0.00011
175		2	1			3		1					4	2	1	1			3	2	12	4			4	1		3	44	0.00008
177	3	2		1	4	1	3	1	5	4	1	1	7	3	2	12	5	2	1	2	1	5		1	10	14	4	4	99	0.00017
178	3	6			4	1	3			1	1	1	4		2	2	3	2		3		4		1	5	5		5	56	0.00010
182	2	5		1	1	5	5			1	2	2	2		5	4	3	1	2	1				1	1	1	1		46	0.00008
185	4	2	1			3	3	2	2		2	2	2	3	1	2	5	1	1	5	1				3	5		1	51	0.00009
186	3					5	3	1	1		2	2		2	2	1	4	1	1	3	7		1		5		1		45	0.00008
188	2	1			1		1	1	1			2	5		2		2	3	1	2				1	8	6		3	42	0.00007
194	3				4	1		1			3		2	2	2	4	1	3					1	4	3	7		2	43	0.00007
195	1	2		1	1		1	3		1			1	1	5			6		3			3	2	1	5		1	38	0.00007
199	14	10	3	5	15	11	34	10	5	6	4	13	26	9	17	11	17	13	6	10	16	4	3	4	18	27	3	17	331	0.00057
204	5	2			1	1	3		2		1		3	1	4	3	3	1				1	2		2	11		1	47	0.00008
205	3	2		2	1	2	3			1		1	6		2	3	4	1	3	1		1			2	3	2	3	46	0.00008
206	9	6	1	11	8	6	4	1	4	1	3	6	11	5	6	11	9	11	2	8		6	5	6	14	21	1	18	194	0.00033
209	4	1			3	1	6	1	3			1	1	3			2	1		5	4		2		4	4	1	2	49	0.00008
210	1	3		2			8	2	1	1	1		6	2	1	2		3	1			4		1	3	13		8	63	0.00011
212	3			5			2	3					1	2	2	8		3	1	2		2	2	1	2	3		5	47	0.00008
218	1			1	1	1	3		1		5	3	1		5	2	5	1		2			1	2	1	12		5	53	0.00009
220	3	2	2		3	3	5	1	2				2	2	1	2	4	1		4		2		2	4	1	1	6	53	0.00009
221	11	4	9	5	2	22	6	1	3	1	2	63	4	2	59	8	7	6	1	7		8	4	1	13	131	3	11	394	0.00068
223	4	3	1			2	1				1	2	1	2	8	1	7	1	1	11	1		2	1	4	11			65	0.00011
227	3	3		1		8	6	1	2				6	2	1		2		1	1	1			1		3	3	5	50	0.00009
229		4	1	1		1	8		2	1		2			1	1	1		2	3	2	2	2	1	5	4		5	49	0.00008
230		2	1		9	6	3	2	2				4	2	4	5	1	2	1	2	2		1	2	6	7	1	10	75	0.00013
233	4	6			1	1	8		2	2	1	6	1	2	2	3	3	1	3	1	2	3	1		2	7		1	63	0.00011
234	2	1			2	6	2						5	1	2			2		3	5		1	6	4	5		2	49	0.00008
235	4	2				2	1						1	4	2			1			3	1	1		3	7		4	36	0.00006
236		3		2	1	3	2		1	4	1	2	3	3	1	3	7	4	4	4	3	5		1		6	1	6	70	0.00012
239	1	6		3	4	4	3		2	4	1	4	4	3	2	7	3	3	1	2	3	6	3		6	13	1	9	98	0.00017
240	2	2			1	6			3	1		1		2		1	1	1	1			3		1	4	5	1	1	37	0.00006
242			1																						2				3	0.00001
244						1					1	1		1		2		1				3			1	7		4	22	0.00004
247			1					1					1	1			2								3				9	0.00002
248	2	2	2			1	1			1	1	3	1	2	1	2	2		4	4	3	1	1		6	3		4	47	0.00008
253	2		1		1	4	4		2	1		1	3	2	1	5	3	2			1	2			3	6	1	3	48	0.00008
255			13	1										10			1										2		27	0.00005
258				1						1			1												1				4	0.00001
260			1					1				1			2														5	0.00001
271																			1										1	0.00000
Total	572	472	155	180	324	523	652	187	225	256	161	482	690	421	453	368	443	379	297	541	278	384	142	212	827	1229	107	804	11764	

P-values per position per pool

																													Count			All 3D coordinates	
pos	X1	X2	X3	X4	X5	X6	X7	X8	X9	X10	X11	X12	Y1	Y2	Y3	Y4	Y5	Y6	Y7	Y8	Z1	Z2	Z3	Z4	Z5	Z6	Z7	Z8	P<0.001	P<0.01	P<0.05	0.001	0.01	0.05
26	0.4	0.8	1.0	1.0	0.8	0.9	0.9	0.5	0.7	1.0	0.5	1.0	0.3	0.5	0.2	0.8	1.0	0.8	1.0	0.3	0.6	0.8	1.0	0.0	0.9	0.6	0.3	0.0	0	1	2	0	0	0
27	1.0	1.0	1.0	1.0	0.2	1.0	1.0	1.0	1.0	1.0	1.0	1.0	0.1	1.0	1.0	0.0	1.0	1.0	1.0	0.2	1.0	1.0	1.0	1.0	1.0	1.0	1.0	0.4	0	0	1	0	0	0
30	1.0	1.0	1.0	1.0	0.2	0.3	0.0	1.0	1.0	1.0	1.0	1.0	1.0	1.0	1.0	1.0	1.0	1.0	1.0	1.0	1.0	1.0	1.0	1.0	1.0	1.0	1.0	0.4	1	1	1	0	0	0
32	0.6	0.2	0.4	0.5	0.4	0.1	0.2	1.0	0.6	1.0	1.0	1.0	1.0	1.0	0.2	0.2	0.2	0.4	1.0	1.0	1.0	0.8	0.5	1.0	0.1	0.6	1.0	0.8	0	0	0	0	0	0
33	0.4	1.0	1.0	1.0	1.0	0.4	1.0	1.0	1.0	1.0	1.0	0.3	0.2	1.0	1.0	1.0	0.4	0.3	1.0	1.0	1.0	1.0	1.0	1.0	0.2	1.0	1.0	0.2	0	0	0	0	0	0
37	0.9	0.8	0.4	1.0	0.2	1.0	0.6	0.0	1.0	0.0	1.0	0.1	0.1	0.8	1.0	0.5	0.6	0.2	1.0	1.0	0.6	0.9	1.0	1.0	1.0	0.9	1.0	0.7	1	1	2	0	0	0
40	0.7	0.0	1.0	1.0	1.0	0.1	0.5	1.0	0.8	0.8	1.0	1.0	1.0	1.0	0.5	0.8	1.0	0.3	0.1	0.6	0.8	0.8	1.0	0.4	0.6	0.2	0.5	1.0	1	1	1	0	0	0
41	0.4	0.0	0.8	0.1	0.9	0.5	1.0	0.4	0.7	0.3	0.0	0.0	0.6	1.0	0.9	1.0	1.0	0.2	0.3	0.1	0.9	0.8	0.4	0.1	0.5	0.0	0.0	0.6	2	2	5	0	0	0
43	0.1	0.9	0.4	0.9	0.9	0.6	0.2	0.9	0.1	0.6	0.9	0.8	0.5	0.3	0.9	0.4	0.9	0.7	1.0	0.1	0.6	0.8	0.5	0.9	1.0	0.0	0.6	0.2	0	0	1	0	0	0
44	0.2	0.9	1.0	0.3	0.3	0.4	0.9	0.7	1.0	0.7	1.0	0.3	0.9	0.9	1.0	0.9	1.0	0.2	0.5	0.5	0.7	0.9	1.0	0.7	0.3	0.1	0.4	0.0	0	0	1	0	0	0
46	0.9	0.8	1.0	0.6	0.8	0.9	0.4	0.0	1.0	0.6	0.2	0.5	0.4	0.5	0.3	0.6	0.3	0.0	1.0	0.2	1.0	0.9	0.5	1.0	0.4	0.7	1.0	1.0	0	0	2	0	0	0
48	0.6	0.9	0.2	0.7	0.3	0.8	0.9	0.3	0.8	1.0	0.3	0.3	1.0	0.0	0.4	0.9	0.7	0.6	0.4	0.9	0.7	0.5	0.6	1.0	0.8	0.0	0.4	0.7	0	0	2	0	0	0
51	0.5	0.5	0.7	0.8	0.8	0.9	0.0	0.2	0.9	0.3	0.4	0.1	0.2	1.0	0.1	0.7	0.3	0.8	0.6	0.3	0.5	0.7	1.0	0.8	0.8	0.9	0.5	1.0	0	0	1	0	0	0
54	0.8	0.1	1.0	0.7	0.9	0.6	0.1	1.0	1.0	0.7	0.3	0.9	0.4	1.0	0.9	1.0	0.3	0.9	0.5	0.9	1.0	0.1	1.0	0.3	0.4	1.0	0.1	0.0	1	1	1	0	0	0
57	0.3	0.9	0.6	0.2	1.0	0.0	0.1	0.0	0.3	0.8	1.0	1.0	0.9	0.3	0.1	0.9	0.8	0.3	1.0	0.6	0.2	0.6	1.0	0.0	1.0	0.8	1.0	0.7	0	2	3	0	0	0
58	0.0	0.9	1.0	1.0	0.6	0.6	0.2	1.0	0.5	0.2	1.0	1.0	0.9	0.7	0.4	0.7	0.9	0.9	0.8	1.0	0.1	1.0	0.1	0.3	1.0	0.8	1.0	0.1	1	1	1	0	0	0
59	0.1	0.0	0.6	0.7	1.0	0.7	0.5	0.9	0.7	0.9	0.7	0.6	0.8	0.0	1.0	1.0	0.7	0.6	1.0	0.7	0.7	0.2	1.0	0.0	0.6	0.3	0.7	0.3	1	3	3	0	1	1
60	0.9	0.1	0.1	0.8	1.0	0.3	0.4	0.4	0.3	1.0	1.0	0.2	1.0	0.8	0.9	1.0	0.4	0.9	0.6	0.1	0.2	0.6	1.0	0.7	0.8	0.2	0.2	0.3	0	0	0	0	0	0
61	0.8	0.3	0.2	1.0	0.2	0.7	0.7	1.0	0.1	0.5	0.8	0.4	0.9	0.3	0.1	0.5	0.3	0.9	0.3	0.3	1.0	0.6	1.0	0.4	0.6	0.6	1.0	0.1	0	0	0	0	0	0
62	0.9	0.2	0.2	0.5	0.2	1.0	0.5	0.6	0.9	0.0	0.2	0.6	0.7	0.6	0.8	0.2	0.2	0.2	0.5	0.2	1.0	1.0	0.9	0.1	0.7	0.4	0.9	0.7	0	0	1	0	0	0
63	0.9	0.1	1.0	1.0	0.3	0.0	0.7	0.6	0.8	0.1	1.0	0.1	0.3	0.6	0.0	0.9	1.0	0.3	0.8	0.4	0.3	0.9	1.0	0.6	0.5	1.0	0.4	1.0	0	1	2	0	0	0
64	0.6	0.1	0.3	0.8	0.9	0.8	0.4	0.2	0.9	1.0	0.7	0.7	0.5	0.6	1.0	0.6	0.7	0.9	0.1	0.3	0.0	0.4	0.7	1.0	0.2	1.0	1.0	0.1	0	0	1	0	0	0
67	0.4	1.0	1.0	1.0	0.6	0.9	0.9	0.1	0.8	0.1	1.0	0.6	1.0	0.9	0.6	0.7	0.9	0.2	0.1	0.2	1.0	0.9	0.6	0.3	0.4	0.1	0.4	0.3	0	0	0	0	0	0
68	0.7	0.6	0.2	0.8	0.9	0.5	0.4	0.0	1.0	0.0	0.1	0.0	0.9	0.7	0.0	0.6	0.9	1.0	0.0	0.4	0.0	1.0	1.0	0.9	1.0	1.0	0.8	1.0	2	4	6	0	2	6
70	0.3	0.5	1.0	1.0	0.3	0.9	1.0	0.6	0.7	0.3	1.0	1.0	0.1	0.9	1.0	0.4	0.7	0.9	0.2	0.9	0.3	0.2	0.6	0.6	0.5	0.2	1.0	0.6	0	0	0	0	0	0
71	0.9	0.3	0.5	0.1	0.0	0.8	0.5	0.0	1.0	0.0	1.0	0.3	0.9	1.0	0.0	0.9	0.1	0.7	0.0	0.2	0.0	0.9	1.0	1.0	1.0	1.0	1.0	1.0	0	5	6	0	4	6
73	1.0	0.8	1.0	0.1	1.0	0.3	0.5	0.2	1.0	0.7	0.2	0.3	0.1	0.1	1.0	0.2	0.9	0.9	0.2	0.9	0.7	0.4	1.0	1.0	0.3	0.8	1.0	0.8	0	0	0	0	0	0
74	1.0	0.8	1.0	0.7	0.0	0.5	0.9	0.2	1.0	1.0	1.0	1.0	0.9	0.9	1.0	0.4	0.2	0.1	0.8	0.1	1.0	0.7	0.6	0.6	0.5	0.2	0.4	0.2	0	0	1	0	0	0
76	0.7	0.8	1.0	1.0	0.8	0.9	0.9	1.0	1.0	0.1	1.0	0.2	0.8	0.1	0.2	0.5	1.0	0.8	0.7	0.1	0.1	0.0	1.0	1.0	0.8	0.3	1.0	1.0	0	0	1	0	0	0
77	0.8	0.3	1.0	1.0	0.6	0.8	0.7	1.0	0.1	0.0	1.0	0.8	0.9	0.1	0.9	0.2	0.0	0.6	0.0	0.9	0.7	0.7	1.0	0.6	0.8	0.9	1.0	0.7	0	1	3	0	0	0
79	0.8	1.0	1.0	1.0	0.7	0.3	0.2	1.0	0.1	0.5	0.1	0.1	1.0	1.0	0.7	0.8	1.0	0.1	1.0	0.5	0.2	1.0	1.0	0.5	0.1	0.7	1.0	0.8	0	0	0	0	0	0
81	0.2	1.0	0.2	0.3	0.8	0.7	1.0	0.2	0.7	0.7	1.0	1.0	0.5	1.0	1.0	0.6	0.4	1.0	0.7	0.2	0.7	0.0	1.0	0.6	0.0	0.2	1.0	0.9	0	0	2	0	0	0
82	0.3	0.5	1.0	1.0	0.3	0.7	0.4	1.0	0.4	1.0	1.0	0.1	0.5	0.8	1.0	1.0	0.7	0.0	0.0	1.0	0.7	1.0	0.0	0.6	0.9	1.0	1.0	1.0	2	2	3	0	0	0
85	0.1	0.4	1.0	0.6	1.0	0.9	0.8	0.2	1.0	1.0	1.0	1.0	0.3	0.8	1.0	1.0	0.6	0.8	0.0	0.6	0.6	0.5	0.5	0.0	0.0	1.0	0.3	0.9	0	1	3	0	0	0
87	0.7	0.8	1.0	1.0	0.8	1.0	0.1	1.0	1.0	0.6	1.0	1.0	0.1	0.1	0.8	1.0	0.3	1.0	0.7	0.0	0.6	0.6	1.0	1.0	0.0	0.7	1.0	0.9	0	0	2	0	0	0
88	0.8	0.2	0.6	0.7	1.0	1.0	0.9	0.3	0.8	0.0	1.0	0.9	0.0	0.0	0.4	1.0	0.3	0.1	0.8	0.9	0.7	1.0	0.6	0.3	1.0	0.9	0.4	0.7	1	2	3	0	0	0
91	0.1	0.4	1.0	1.0	1.0	0.8	0.4	1.0	0.6	1.0	1.0	0.1	0.5	0.2	0.7	0.8	0.5	1.0	0.2	0.2	0.6	1.0	1.0	1.0	0.5	0.5	0.0	0.9	0	0	1	0	0	0
94	0.2	0.8	0.5	1.0	1.0	0.4	0.0	1.0	1.0	1.0	1.0	1.0	0.8	0.8	1.0	0.6	0.6	1.0	0.0	1.0	0.3	0.6	1.0	1.0	0.1	0.7	1.0	0.2	0	1	2	0	0	0
97	1.0	0.3	1.0	0.5	0.7	1.0	0.9	1.0	1.0	0.5	0.4	0.3	0.4	0.7	0.1	0.2	0.8	0.0	1.0	0.4	0.2	0.8	1.0	1.0	0.7	0.9	1.0	0.5	0	0	1	0	0	0
100	0.7	0.1	0.5	1.0	1.0	1.0	0.2	1.0	0.7	0.3	0.6	1.0	0.3	0.5	0.8	0.1	0.0	0.8	1.0	0.6	0.1	0.6	1.0	0.2	1.0	0.4	1.0	0.8	0	0	1	0	0	0
103	0.8	0.5	1.0	0.1	0.5	0.7	0.6	0.2	1.0	0.3	1.0	0.2	0.1	0.8	0.3	1.0	0.7	0.8	0.1	0.9	0.3	0.2	1.0	0.6	0.5	0.9	1.0	0.8	0	0	0	0	0	0
104	0.7	0.5	1.0	0.6	0.4	0.9	0.8	0.0	0.1	0.3	0.5	1.0	0.4	0.5	0.5	0.5	0.6	0.8	1.0	0.6	0.3	0.9	0.5	0.5	0.6	0.9	1.0	0.7	0	1	1	0	0	0
106	0.1	0.2	0.0	1.0	0.2	0.4	0.5	1.0	1.0	1.0	1.0	0.6	1.0	0.2	0.6	0.3	0.7	0.6	1.0	1.0	1.0	0.7	1.0	1.0	0.8	0.7	1.0	0.6	0	0	1	0	0	0
112	0.7	0.0	0.4	1.0	0.4	0.9	0.8	1.0	0.3	1.0	1.0	0.0	0.3	0.1	0.1	1.0	1.0	0.5	1.0	1.0	0.6	0.3	1.0	0.0	1.0	1.0	1.0	1.0	1	1	3	0	0	0
115	0.9	0.0	0.4	1.0	1.0	0.9	0.3	1.0	1.0	1.0	1.0	0.2	0.3	0.5	0.8	0.8	0.1	0.8	1.0	0.0	0.6	0.8	1.0	1.0	0.6	0.7	1.0	1.0	1	1	2	0	0	0
118	1.0	0.7	0.1	0.5	0.7	0.8	0.7	1.0	0.6	0.2	1.0	0.1	0.9	0.4	0.0	0.4	0.2	0.4	1.0	0.8	0.5	1.0	1.0	1.0	0.7	0.5	1.0	0.8	0	0	1	0	0	0
122	1.0	0.6	1.0	0.1	1.0	0.2	0.9	1.0	1.0	1.0	1.0	1.0	0.9	0.7	0.3	0.0	0.4	0.7	0.2	0.7	1.0	0.7	1.0	1.0	0.2	0.4	0.2	0.9	0	0	1	0	0	0
127	1.0	0.5	1.0	1.0	1.0	1.0	0.0	0.0	1.0	0.4	1.0	0.5	1.0	0.6	0.6	0.2	1.0	0.6	0.4	1.0	0.4	0.6	1.0	0.1	0.8	0.9	1.0	0.8	0	1	2	0	0	0
131	0.0	0.2	1.0	0.9	1.0	0.0	1.0	0.2	0.7	1.0	0.4	0.5	1.0	0.4	0.3	0.3	0.9	0.4	0.5	1.0	0.6	0.7	0.1	0.1	0.4	0.3	1.0	0.6	0	1	2	0	0	0
132	1.0	0.1	1.0	0.7	0.3	0.5	0.5	0.6	0.8	0.4	1.0	0.8	1.0	0.1	0.2	0.9	0.7	0.4	0.8	0.9	0.4	0.7	0.2	0.6	0.1	0.9	1.0	0.5	0	0	0	0	0	0
133	0.0	0.3	0.1	1.0	0.4	0.0	0.0	0.9	0.9	0.8	0.6	0.8	0.0	0.2	0.9	1.0	0.0	0.8	0.9	0.9	0.0	0.0	0.8	0.1	0.0	0.0	0.1	0.0	9	10	10	24	30	30
134	0.9	1.0	0.1	1.0	1.0	0.7	0.6	0.2	1.0	0.6	1.0	1.0	0.2	0.1	0.5	0.9	0.0	0.8	0.7	0.9	1.0	1.0	1.0	1.0	0.2	0.1	1.0	0.5	0	1	1	0	0	0
137	0.3	0.1	1.0	1.0	1.0	1.0	1.0	0.6	0.8	1.0	0.2	0.1	0.9	0.9	1.0	0.4	0.2	0.9	0.8	0.2	1.0	1.0	0.6	1.0	0.0	0.5	1.0	0.1	0	1	1	0	0	0
138	0.4	1.0	0.7	0.5	0.3	0.6	0.1	0.4	0.6	0.0	1.0	1.0	0.5	0.6	0.8	0.8	0.9	0.6	0.6	0.7	0.5	0.7	0.7	1.0	0.5	0.9	1.0	0.2	0	1	1	0	0	0
145	0.9	0.1	1.0	0.6	0.8	1.0	1.0	1.0	0.1	0.0	1.0	1.0	0.6	0.1	0.1	0.6	0.2	0.8	1.0	0.6	0.7	0.1	1.0	1.0	0.7	0.1	1.0	1.0	0	0	1	0	0	0
146	1.0	1.0	1.0	1.0	1.0	0.1	0.0	0.5	0.7	1.0	1.0	1.0	0.2	0.0	0.8	0.6	0.9	0.5	0.3	0.9	0.3	1.0	1.0	1.0	1.0	0.0	1.0	0.7	0	1	3	0	0	1
147	0.7	0.5	0.5	1.0	0.5	0.7	1.0	0.2	0.4	0.0	0.0	0.5	0.2	0.5	0.5	1.0	0.9	0.8	0.1	0.6	1.0	0.9	0.2	1.0	0.3	1.0	1.0	0.6	0	0	2	0	0	0
151	0.8	1.0	1.0	1.0	0.3	0.1	0.9	0.1	0.2	1.0	1.0	0.6	0.3	1.0	0.7	0.3	1.0	0.7	1.0	0.7	0.1	0.7	0.4	1.0	0.6	1.0	0.2	0.4	0	0	0	0	0	0
156	0.0	0.8	0.5	1.0	0.8	0.0	1.0	1.0	0.7	1.0	0.2	1.0	0.0	1.0	0.3	0.9	0.9	0.8	1.0	0.9	0.6	0.9	1.0	1.0	0.4	1.0	0.0	0.7	0	3	4	0	1	2
158	1.0	0.0	1.0	1.0	0.6	0.5	0.4	0.8	0.7	1.0	0.2	0.8	0.7	0.0	0.6	0.9	0.9	0.8	1.0	0.6	0.9	1.0	0.4	0.2	0.7	0.5	0.6	0.7	1	1	2	0	0	0
160	0.2	0.6	0.2	0.3	0.3	0.1	0.9	0.6	1.0	1.0	0.2	0.8	0.9	0.9	0.9	0.9	0.7	0.9	0.4	0.2	0.3	0.9	1.0	0.6	0.5	0.4	0.4	0.5	0	0	0	0	0	0
162	0.4	0.8	1.0	1.0	0.8	0.6	1.0	0.5	0.7	0.6	1.0	1.0	0.9	0.4	0.4	0.8	0.1	0.2	1.0	0.5	1.0	1.0	1.0	0.5	0.8	0.0	1.0	0.4	0	1	1	0	0	0
165	0.4	0.9	0.3	0.8	0.7	1.0	0.5	0.7	0.9	0.2	1.0	0.1	0.8	0.8	0.1	0.6	1.0	1.0	1.0	0.1	0.5	0.2	0.7	0.7	0.6	0.0	0.5	1.0	0	0	1	0	0	0
166	0.8	0.1	1.0	0.1	0.1	0.2	0.9	1.0	1.0	0.2	1.0	1.0	0.9	1.0	1.0	1.0	0.2	0.0	0.2	0.4	0.2	0.8	0.4	1.0	0.9	0.9	1.0	0.7	0	0	1	0	0	0
169	0.9	1.0	0.5	0.3	1.0	0.1	0.6	0.6	0.4	0.0	0.6	1.0	0.1	0.3	1.0	0.1	1.0	0.1	0.7	0.6	0.7	1.0	1.0	1.0	0.4	0.7	1.0	0.9	0	1	1	0	0	0
170	1.0	1.0	0.8	1.0	1.0	1.0	1.0	1.0	1.0	0.4	1.0	0.0	1.0	1.0	1.0	1.0	1.0	1.0	0.0	0.0	1.0	1.0	1.0	0.7	0.0	1.0	1.0	1.0	4	4	4	2	2	2
174	0.8	0.0	1.0	0.1	0.9	0.5	0.5	0.3	0.8	0.1	1.0	0.3	0.7	0.6	0.3	0.7	0.7	0.9	0.8	0.1	1.0	1.0	0.2	1.0	0.8	0.7	1.0	0.8	0	1	1	0	0	0
175	1.0	0.4	0.4	1.0	1.0	0.3	1.0	0.5	1.0	1.0	1.0	1.0	0.3	0.4	0.8	0.8	1.0	1.0	0.1	0.5	0.0	0.1	1.0	1.0	0.3	1.0	1.0	0.6	1	1	1	0	0	0
177	0.9	0.8	1.0	0.8	0.4	1.0	0.9	0.8	0.1	0.1	0.8	1.0	0.4	0.7	0.9	0.0	0.4	0.9	0.9	0.9	0.9	0.4	1.0	0.8	0.1	0.1	0.0	0.9	1	1	2	0	0	0
178	0.5	0.0	1.0	1.0	0.1	0.9	0.7	1.0	1.0	0.7	0.6	0.8	0.5	1.0	0.6	0.6	0.4	0.6	1.0	0.4	1.0	0.2	1.0	0.6	0.3	0.6	1.0	0.4	0	0	1	0	0	0
182	0.7	0.0	1.0	0.6	0.8	0.1	0.1	1.0	1.0	0.6	0.2	0.4	0.8	1.0	0.0	0.1	0.3	0.8	0.3	0.8	1.0	1.0	1.0	0.5	0.9	1.0	0.3	1.0	0	0	2	0	0	0
185	0.2	0.5	0.5	1.0	1.0	0.4	0.6	0.2	0.3	1.0	0.2	0.5	0.8	0.3	0.8	0.6	0.1	0.8	0.7	0.1	0.6	1.0	1.0	1.0	0.6	0.5	1.0	1.0	0	0	0	0	0	0
186	0.4	1.0	1.0	1.0	1.0	0.1	0.5	0.5	0.7	1.0	0.2	0.4	1.0	0.5	0.5	0.8	0.1	0.8	0.7	0.3	0.0	1.0	0.5	1.0	0.2	1.0	0.3	1.0	1	1	1	0	0	0
188	0.6	0.7	1.0	1.0	0.7	1.0	0.9	0.5	0.6	1.0	1.0	0.4	0.1	1.0	0.4	1.0	0.5	0.2	0.6	0.5	1.0	1.0	1.0	0.5	0.0	0.2	1.0	0.6	0	1	1	0	0	0
194	0.4	1.0	1.0	1.0	0.0	0.9	1.0	0.5	1.0	1.0	0.0	1.0	0.8	0.4	0.4	0.1	0.8	0.2	1.0	1.0	1.0	1.0	0.5	0.0	0.5	0.1	1.0	0.8	0	1	3	0	0	0
195	0.8	0.3	1.0	0.5	0.7	1.0	0.9	0.0	1.0	0.5	1.0	1.0	0.9	0.7	0.0	1.0	1.0	0.0	1.0	0.2	1.0	1.0	0.0	0.1	0.9	0.3	1.0	0.9	0	1	4	0	0	2
199	0.7	0.6	0.8	0.7	0.1	0.9	0.0	0.0	0.9	0.7	0.8	0.2	0.2	0.8	0.1	0.7	0.2	0.3	0.8	0.9	0.0	1.0	0.9	0.8	0.8	0.8	0.5	1.0	0	2	3	0	0	0
204	0.1	0.4	1.0	1.0	0.8	0.9	0.5	1.0	0.3	1.0	0.5	1.0	0.6	0.8	0.1	0.3	0.3	0.8	1.0	1.0	1.0	0.8	0.2	1.0	0.8	0.0	1.0	1.0	0	1	1	0	0	0
205	0.4	0.4	1.0	0.2	0.8	0.6	0.5	1.0	1.0	0.6	1.0	0.8	0.1	1.0	0.5	0.3	0.1	0.8	0.1	0.8	1.0	0.8	1.0	1.0	0.8	0.8	0.1	0.7	0	0	0	0	0	0
206	0.6	0.6	0.9	0.0	0.3	0.9	1.0	1.0	0.7	1.0	0.6	0.5	0.7	0.8	0.7	0.1	0.4	0.1	0.9	0.5	1.0	0.8	0.2	0.1	0.4	0.3	0.8	0.2	0	1	1	0	0	0
209	0.2	0.8	1.0	1.0	0.2	0.9	0.1	0.5	0.1	1.0	1.0	0.8	1.0	0.2	1.0	1.0	0.6	0.8	1.0	0.1	0.0	1.0	0.2	1.0	0.4	0.7	0.3	0.9	0	0	1	0	0	0
210	1.0	0.3	1.0	0.3	1.0	1.0	0.0	0.3	0.8	0.7	0.6	1.0	0.2	0.6	0.9	0.7	1.0	0.4	0.8	1.0	1.0	0.2	1.0	0.6	0.8	0.0	1.0	0.1	0	1	2	0	0	0
212	0.4	1.0	1.0	0.0	1.0	1.0	0.8	0.0	1.0	1.0	1.0	1.0	0.9	0.5	0.5	0.0	1.0	0.2	0.7	0.6	1.0	0.6	0.2	0.5	0.8	0.8	1.0	0.3	1	2	3	0	0	0
218	0.9	1.0	1.0	0.6	0.8	0.9	0.6	1.0	0.7	1.0	0.0	0.2	1.0	1.0	0.0	0.6	0.1	0.8	1.0	0.6	1.0	1.0	0.5	0.2	1.0	0.0	1.0	0.4	0	2	3	0	0	1
220	0.5	0.5	0.1	1.0	0.2	0.4	0.2	0.6	0.4	1.0	1.0	1.0	0.8	0.5	0.8	0.6	0.2	0.8	1.0	0.2	1.0	0.6	1.0	0.2	0.4	1.0	0.4	0.2	0	0	0	0	0	0
221	1.0	1.0	0.1	0.9	1.0	0.2	1.0	1.0	1.0	1.0	1.0	0.0	1.0	1.0	0.0	1.0	1.0	1.0	1.0	1.0	1.0	1.0	0.8	1.0	1.0	0.0	0.6	1.0	3	3	3	1	1	1
223	0.4	0.3	0.6	1.0	1.0	0.8	1.0	1.0	1.0	1.0	0.6	0.6	1.0	0.6	0.0	0.9	0.0	0.9	0.8	0.0	0.7	1.0	0.3	0.6	0.6	0.1	1.0	1.0	1	2	3	0	0	0
227	0.4	0.2	1.0	0.6	1.0	0.0	0.1	0.5	0.3	1.0	1.0	1.0	0.1	0.5	0.8	1.0	0.6	1.0	0.7	0.9	0.6	1.0	1.0	0.6	1.0	0.9	0.0	0.3	0	2	2	0	0	0
229	1.0	0.1	0.5	0.6	1.0	0.9	0.0	1.0	0.3	0.6	1.0	0.4	1.0	1.0	0.8	0.8	0.9	1.0	0.3	0.3	0.3	0.6	0.2	0.5	0.2	0.7	1.0	0.3	0	0	1	0	0	0
230	1.0	0.7	0.6	1.0	0.0	0.1	0.8	0.3	0.5	1.0	1.0	1.0	0.7	0.7	0.3	0.2	1.0	0.7	0.8	0.8	0.5	1.0	0.7	0.3	0.4	0.6	0.5	0.1	1	1	1	0	0	0
233	0.4	0.0	1.0	1.0	0.9	0.9	0.0	1.0	0.4	0.4	0.6	0.0	1.0	0.6	0.6	0.4	0.5	0.9	0.2	0.9	0.4	0.5	0.6	1.0	0.9	0.4	1.0	1.0	0	0	3	0	0	0
234	0.7	0.8	1.0	1.0	0.5	0.0	0.8	1.0	1.0	1.0	1.0	1.0	0.2	0.8	0.5	1.0	1.0	0.5	1.0	0.3	0.0	1.0	0.5	0.0	0.4	0.5	1.0	0.9	1	2	3	0	0	0
235	0.1	0.3	1.0	1.0	1.0	0.5	0.9	1.0	1.0	1.0	1.0	1.0	0.9	0.0	0.4	1.0	1.0	0.7	1.0	1.0	0.0	0.8	0.4	1.0	0.4	0.1	1.0	0.3	0	0	2	0	0	0
236	1.0	0.4	1.0	0.4	0.9	0.6	0.9	1.0	0.8	0.1	0.7	0.6	0.8	0.4	0.9	0.5	0.0	0.2	0.1	0.3	0.2	0.1	1.0	0.7	1.0	0.7	0.4	0.4	0	0	1	0	0	0
239	1.0	0.1	1.0	0.3	0.4	0.7	0.9	1.0	0.7	0.1	0.8	0.3	0.9	0.6	0.8	0.1	0.8	0.6	0.9	0.9	0.3	0.2	0.2	1.0	0.6	0.2	0.6	0.3	0	0	0	0	0	0
240	0.5	0.3	1.0	1.0	0.7	0.0	1.0	1.0	0.1	0.5	1.0	0.7	1.0	0.4	1.0	0.8	0.8	0.7	0.6	1.0	1.0	0.2	1.0	0.4	0.2	0.3	0.3	0.9	0	1	1	0	0	0
242	1.0	1.0	0.0	1.0	1.0	1.0	1.0	1.0	1.0	1.0	1.0	1.0	1.0	1.0	1.0	1.0	1.0	1.0	1.0	1.0	1.0	1.0	1.0	1.0	0.0	1.0	1.0	1.0	0	0	2	0	0	0
244	1.0	1.0	1.0	1.0	1.0	0.6	1.0	1.0	1.0	1.0	0.3	0.5	1.0	0.5	1.0	0.2	1.0	0.5	1.0	1.0	1.0	0.1	1.0	1.0	0.8	0.0	1.0	0.1	0	1	1	0	0	0
247	1.0	1.0	0.1	1.0	1.0	1.0	1.0	0.1	1.0	1.0	1.0	1.0	0.4	0.3	1.0	1.0	0.1	1.0	1.0	1.0	1.0	1.0	1.0	1.0	0.0	1.0	1.0	1.0	0	0	1	0	0	0
248	0.7	0.4	0.1	1.0	1.0	0.9	0.9	1.0	1.0	0.6	0.5	0.2	0.9	0.5	0.8	0.5	0.6	1.0	0.0	0.1	0.1	0.8	0.5	1.0	0.1	0.8	1.0	0.5	0	0	1	0	0	0
253	0.7	1.0	0.4	1.0	0.8	0.2	0.3	1.0	0.3	0.6	1.0	0.8	0.6	0.5	0.8	0.0	0.3	0.5	1.0	1.0	0.6	0.6	1.0	1.0	0.6	0.3	0.3	0.7	0	0	1	0	0	0
255	1.0	1.0	0.0	0.4	1.0	1.0	1.0	1.0	1.0	1.0	1.0	1.0	1.0	0.0	1.0	1.0	0.7	1.0	1.0	1.0	1.0	1.0	1.0	1.0	1.0	1.0	0.0	1.0	2	2	3	0	0	1
258	1.0	1.0	1.0	0.1	1.0	1.0	1.0	1.0	1.0	0.1	1.0	1.0	0.2	1.0	1.0	1.0	1.0	1.0	1.0	1.0	1.0	1.0	1.0	1.0	0.2	1.0	1.0	1.0	0	0	0	0	0	0
260	1.0	1.0	0.1	1.0	1.0	1.0	1.0	0.1	1.0	1.0	1.0	0.1	1.0	1.0	0.0	1.0	1.0	1.0	1.0	1.0	1.0	1.0	1.0	1.0	1.0	1.0	1.0	1.0	0	0	1	0	0	0
271	1.0	1.0	1.0	1.0	1.0	1.0	1.0	1.0	1.0	1.0	1.0	1.0	1.0	1.0	1.0	1.0	1.0	1.0	0.0	1.0	1.0	1.0	1.0	1.0	1.0	1.0	1.0	1.0	0	0	1	0	0	0
