# Supplementary material for: High-Throughput Detection of Induced Mutations and Natural Variation Using KeyPoint™ Technology
Source: PLoS One. 2009 Mar 13;4(3):e4761. doi: 10.1371/journal.pone.0004761 (PMC2654077; doi:10.1371/journal.pone.0004761)
Supplement: Figure S5 — Results KeyPoint analysis on natural populations. (0.03 MB PDF) [file pone.0004761.s005.pdf]

A. KeyPoint NVS analysis - amplicon 1

Observed counts per position per pool, when more than 2 polymorphisms per position

Sums per pool

			###	627	832	955	###	###	###	738	###	###	###	###	###	###	###	###	###	###	###	###		
pos	WT	SNP	X1	X2	X3	X4	X5	X6	X7	X8	X9	X10	X11	X12	Y1	Y2	Y3	Y4	Y5	Y6	Y7	Y8	Total	error rate
26	C	T	1			1									1			1	1				5	0.00016
32	C	T						2	1							2						1	6	0.00019
40	G	A	2												1			1					4	0.00013
41	C	T	10	1	3	4	2	5	2	1	4	1		3	5	5	6	3	2	5	12	5	79	0.00249
43	G	A	2				1	2	3	1					1	1			1			1	13	0.00041
44	C	A					1										1					1	3	0.00009
45	T	C	3					2					1						1	1	2		10	0.00032
48	G	A					2	1														1	4	0.00013
48	G	T					1		1			1						1					4	0.00013
49	A	T	1		2										1				1				5	0.00016
51	G	A	1		2		1	1				1	2		1				1				10	0.00032
52	T	G	1		2										1				1				5	0.00016
53	T	A				1	1		2		3		1			2		2			2	1	15	0.00047
55	A	G			1				3									1			1		6	0.00019
56	A	C	3			1	1		3	1	1		1	1	1	1		2	2			1	19	0.00060
56	A	G	1	1	1	1		1	1		1				1		1						9	0.00028
56	A	T	1		1				2					1	1	1		1			3		11	0.00035
57	G	T					2	1												1	1		5	0.00016
58	G	A	2					1	1								1			1	1		7	0.00022
59	C	A					1	1	2		2	1	2		2	1				2			14	0.00044
59	C	T	6	1			4	1	6		1	2	2		2	1				1	2	1	30	0.00095
60	C	G					1								1		1				2		5	0.00016
61	G	A	1	3			1	2		2	3	1			1	3		2		1		1	21	0.00066
61	G	C			1												1				1		3	0.00009
61	G	T						2								1						1	4	0.00013
62	C	A	1						1	1						1		2					6	0.00019
62	C	T	2		1			1	3		1							1	1		1	2	13	0.00041
63	C	A	2			1			2														5	0.00016
63	C	G	1	1													1				2		5	0.00016
63	C	T						1	1						1								3	0.00009
64	G	A	1	1					1		2						1	1	1		1		9	0.00028
64	G	T													3		1	1					5	0.00016
65	A	G	2	1					1									1			1		6	0.00019
66	T	C							1	1		1	1		2	1				1	2		10	0.00032
67	G	T							1	1		1	1	1	2					1	1	1	10	0.00032
68	G	A							2			1					1		1				5	0.00016
69	A	G	1	1				2	1			1			1	1	1	1	1	3	2	1	17	0.00054
69	A	T	1					1			2				1	1		1			1		8	0.00025
70	G	A	1			1		1					2		3	1	1						10	0.00032
71	G	A	1									2					1		1			1	6	0.00019
73	G	A	1												2	1	2						6	0.00019
73	G	T										1				2							3	0.00009
74	G	A	2												1							2	5	0.00016
74	G	T	3							1										1			5	0.00016
75	A	G	1	1											1	2	1				1	1	8	0.00025
78	A	G	1						1						1								3	0.00009
79	G	T	2												1								3	0.00009
86	A	G													2						1		3	0.00009
88	G	A	1																			3	4	0.00013
91	G	A									2										1		3	0.00009
94	C	A	1							1	1										1		4	0.00013
94	C	T													1				1			1	3	0.00009
96	T	A	1								1			1		1					1		5	0.00016
100	G	A			1						1				1	1							4	0.00013
101	A	G			1										2								3	0.00009
104	G	T												2		1					1		4	0.00013
105	T	A												1		1					1		3	0.00009
105	T	C	1		1		1							1									4	0.00013
106	G	A						1							1		1					1	4	0.00013
107	A	T									1										1	1	3	0.00009
115	G	A																2			1		3	0.00009
118	G	A			1			1								1							3	0.00009
126	T	A					1				3												4	0.00013
127	G	A	1									2				2							5	0.00016
131	C	T										1					1				1		3	0.00009
133	G	A	16	4	3	5	5	10	9	3	4	4	4	2	8	9	6	15	3	8	6	10	134	0.00422
134	C	A	2														1			1			4	0.00013
137	C	T											1			1						4	6	0.00019
138	G	A								1				1				1					3	0.00009
144	A	G							1	1	1					1					1	1	6	0.00019
146	G	A							1								3						4	0.00013
147	G	A	2			1																	3	0.00009
148	A	G									1				1				1	3		1	7	0.00022
150	A	C											4										4	0.00013
150	A	G	2																		1		3	0.00009
156	C	A	2												1		1						4	0.00013
157	A	G	2									1			1					1		1	6	0.00019
158	C	T			1	1	1		1						2			1					7	0.00022
160	G	A										145	2					1		161			309	0.00974
161	T	A										145	2							161			308	0.00971
162	G	A										2			2	1							5	0.00016
164	A	G											3										3	0.00009
165	G	A						1				1				1						1	4	0.00013
166	C	T		1						1			1			1				1			5	0.00016
170	C	T	5			1	2	1	1		2	1					1						14	0.00044
172	T	G		211		108		1		93					2			202	122	150		192	1081	0.03408
174	G	A			3			1				2											6	0.00019
176	A	G	1		1						1							1				1	5	0.00016
177	G	A					1	2	1						1					1		2	8	0.00025
179	A	T	2												1								3	0.00009
185	G	A			1										1		1						3	0.00009
186	G	A	1					1							1								3	0.00009
188	C	A	2								1												3	0.00009
188	C	T						1							1						1	2	5	0.00016
193	T	C										1									1	1	3	0.00009
194	G	A			1			1							3	2					1		8	0.00025
194	G	T	9		1	1	3	2	8	2	2		3	3	3	1	3	5	1	4	1	6	58	0.00183
195	G	A							1	1						1							3	0.00009
195	G	T					1		1		2					2						1	7	0.00022
196	T	G	9	1	1	1	3		9	2	1		4	3	3	1	3	4	1	4	1	6	57	0.00180
198	T	G	3	1	3	1	2	3	17	1	5	2	5	5	12	5	6	5	1	3	2	16	98	0.00309
199	G	A	3	1	2	1	1	3	18	1	3	2	5	5	11	4	6	4		2	1	14	87	0.00274
199	G	T	1		1			2			2				3	2		1	1	1	1	4	19	0.00060
200	A	C	3	1	2	1	1		12	1	3	1	5	3	8	4	4	1		2	1	11	64	0.00202

P-values per position per pool

															A	B	C	D	E	F	G	H		Count	
pos	WT	SNP	X1	X2	X3	X4	X5	X6	X7	X8	X9	X10	X11	X12	Y1	Y2	Y3	Y4	Y5	Y6	Y7	Y8	P<0.001	P<0.01	P<0.05
26	C	T	0.5	1.0	1.0	0.1	1.0	1.0	1.0	1.0	1.0	1.0	1.0	1.0	0.3	1.0	1.0	0.3	0.1	1.0	1.0	1.0	0	0	0
32	C	T	1.0	1.0	1.0	1.0	1.0	0.1	0.4	1.0	1.0	1.0	1.0	1.0	1.0	0.0	1.0	1.0	1.0	1.0	1.0	0.4	0	0	1
40	G	A	0.1	1.0	1.0	1.0	1.0	1.0	1.0	1.0	1.0	1.0	1.0	1.0	0.3	1.0	1.0	0.2	1.0	1.0	1.0	1.0	0	0	0
41	C	T	0.6	0.8	0.3	0.2	0.9	0.7	1.0	0.8	0.4	0.9	1.0	0.5	0.7	0.4	0.1	0.9	0.7	0.4	0.0	0.7	1	1	1
43	G	A	0.5	1.0	1.0	1.0	0.5	0.2	0.1	0.3	1.0	1.0	1.0	1.0	0.6	0.5	1.0	1.0	0.3	1.0	1.0	0.6	0	0	0
44	C	A	1.0	1.0	1.0	1.0	0.1	1.0	1.0	1.0	1.0	1.0	1.0	1.0	1.0	1.0	0.1	1.0	1.0	1.0	1.0	0.2	0	0	0
45	T	C	0.1	1.0	1.0	1.0	1.0	0.2	1.0	1.0	1.0	1.0	0.3	1.0	1.0	1.0	1.0	1.0	0.3	0.4	0.1	1.0	0	0	0
48	G	A	1.0	1.0	1.0	1.0	0.0	0.2	1.0	1.0	1.0	1.0	1.0	1.0	1.0	1.0	1.0	1.0	1.0	1.0	1.0	0.3	0	0	1
48	G	T	1.0	1.0	1.0	1.0	0.2	1.0	0.3	1.0	1.0	0.1	1.0	1.0	1.0	1.0	1.0	0.2	1.0	1.0	1.0	1.0	0	0	0
49	A	T	0.5	1.0	0.0	1.0	1.0	1.0	1.0	1.0	1.0	1.0	1.0	1.0	0.3	1.0	1.0	1.0	0.1	1.0	1.0	1.0	0	1	1
51	G	A	0.7	1.0	0.0	1.0	0.4	0.5	1.0	1.0	1.0	0.3	0.0	1.0	0.5	1.0	1.0	1.0	0.3	1.0	1.0	1.0	0	0	2
52	T	G	0.5	1.0	0.0	1.0	1.0	1.0	1.0	1.0	1.0	1.0	1.0	1.0	0.3	1.0	1.0	1.0	0.1	1.0	1.0	1.0	0	1	1
53	T	A	1.0	1.0	1.0	0.4	0.5	1.0	0.3	1.0	0.0	1.0	0.4	1.0	1.0	0.2	1.0	0.3	1.0	1.0	0.1	0.7	0	0	1
55	A	G	1.0	1.0	0.1	1.0	1.0	1.0	0.0	1.0	1.0	1.0	1.0	1.0	1.0	1.0	1.0	0.3	1.0	1.0	0.2	1.0	0	0	1
56	A	C	0.4	1.0	1.0	0.4	0.6	1.0	0.2	0.4	0.6	1.0	0.5	0.5	0.8	0.6	1.0	0.4	0.1	1.0	1.0	0.8	0	0	0
56	A	G	0.7	0.2	0.2	0.2	1.0	0.5	0.5	1.0	0.3	1.0	1.0	1.0	0.5	1.0	0.3	1.0	1.0	1.0	1.0	1.0	0	0	0
56	A	T	0.8	1.0	0.3	1.0	1.0	1.0	0.2	1.0	1.0	1.0	1.0	0.3	0.6	0.4	1.0	0.5	1.0	1.0	0.0	1.0	0	0	1
57	G	T	1.0	1.0	1.0	1.0	0.0	0.3	1.0	1.0	1.0	1.0	1.0	1.0	1.0	1.0	1.0	1.0	1.0	0.2	0.2	1.0	0	0	1
58	G	A	0.2	1.0	1.0	1.0	1.0	0.4	0.4	1.0	1.0	1.0	1.0	1.0	1.0	1.0	0.2	1.0	1.0	0.3	0.2	1.0	0	0	0
59	C	A	1.0	1.0	1.0	1.0	0.5	0.6	0.3	1.0	0.1	0.4	0.1	1.0	0.3	0.5	1.0	1.0	1.0	0.2	1.0	1.0	0	0	0
59	C	T	0.2	0.4	1.0	1.0	0.1	0.9	0.0	1.0	0.7	0.3	0.3	1.0	0.7	0.8	1.0	1.0	1.0	0.8	0.3	0.9	0	0	1
60	C	G	1.0	1.0	1.0	1.0	0.2	1.0	1.0	1.0	1.0	1.0	1.0	1.0	0.3	1.0	0.2	1.0	1.0	1.0	0.0	1.0	0	0	1
61	G	A	0.9	0.0	1.0	1.0	0.7	0.4	1.0	0.1	0.1	0.5	1.0	1.0	0.8	0.1	1.0	0.4	1.0	0.7	1.0	0.8	0	1	1
61	G	C	1.0	1.0	0.1	1.0	1.0	1.0	1.0	1.0	1.0	1.0	1.0	1.0	1.0	1.0	0.1	1.0	1.0	1.0	0.1	1.0	0	0	0
61	G	T	1.0	1.0	1.0	1.0	1.0	0.0	1.0	1.0	1.0	1.0	1.0	1.0	1.0	0.2	1.0	1.0	1.0	1.0	1.0	0.3	0	0	1
62	C	A	0.5	1.0	1.0	1.0	1.0	1.0	0.4	0.1	1.0	1.0	1.0	1.0	1.0	0.3	1.0	0.1	1.0	1.0	1.0	1.0	0	0	0
62	C	T	0.5	1.0	0.3	1.0	1.0	0.6	0.1	1.0	0.4	1.0	1.0	1.0	1.0	1.0	1.0	0.6	0.3	1.0	0.4	0.2	0	0	0
63	C	A	0.1	1.0	1.0	0.1	1.0	1.0	0.1	1.0	1.0	1.0	1.0	1.0	1.0	1.0	1.0	1.0	1.0	1.0	1.0	1.0	0	0	0
63	C	G	0.5	0.1	1.0	1.0	1.0	1.0	1.0	1.0	1.0	1.0	1.0	1.0	1.0	1.0	0.2	1.0	1.0	1.0	0.0	1.0	0	0	1
63	C	T	1.0	1.0	1.0	1.0	1.0	0.2	0.2	1.0	1.0	1.0	1.0	1.0	0.2	1.0	1.0	1.0	1.0	1.0	1.0	1.0	0	0	0
64	G	A	0.7	0.2	1.0	1.0	1.0	1.0	0.5	1.0	0.1	1.0	1.0	1.0	1.0	1.0	0.3	0.5	0.3	1.0	0.3	1.0	0	0	0
64	G	T	1.0	1.0	1.0	1.0	1.0	1.0	1.0	1.0	1.0	1.0	1.0	1.0	0.0	1.0	0.2	0.3	1.0	1.0	1.0	1.0	0	1	1
65	A	G	0.2	0.1	1.0	1.0	1.0	1.0	0.4	1.0	1.0	1.0	1.0	1.0	1.0	1.0	1.0	0.3	1.0	1.0	0.2	1.0	0	0	0
66	T	C	1.0	1.0	1.0	1.0	1.0	1.0	0.5	0.2	1.0	0.3	0.3	1.0	0.2	0.4	1.0	1.0	1.0	0.4	0.1	1.0	0	0	0
67	G	T	1.0	1.0	1.0	1.0	1.0	1.0	0.5	0.2	1.0	0.3	0.3	0.3	0.2	1.0	1.0	1.0	1.0	0.4	0.3	0.5	0	0	0
68	G	A	1.0	1.0	1.0	1.0	1.0	1.0	0.1	1.0	1.0	0.2	1.0	1.0	1.0	1.0	0.2	1.0	0.1	1.0	1.0	1.0	0	0	0
69	A	G	0.9	0.3	1.0	1.0	1.0	0.3	0.7	1.0	1.0	0.5	1.0	1.0	0.7	0.6	0.5	0.7	0.4	0.1	0.2	0.7	0	0	0
69	A	T	0.6	1.0	1.0	1.0	1.0	0.4	1.0	1.0	0.0	1.0	1.0	1.0	0.4	0.3	1.0	0.4	1.0	1.0	0.3	1.0	0	0	1
70	G	A	0.7	1.0	1.0	0.3	1.0	0.5	1.0	1.0	1.0	1.0	0.0	1.0	0.0	0.4	0.3	1.0	1.0	1.0	1.0	1.0	0	0	2
71	G	A	0.5	1.0	1.0	1.0	1.0	1.0	1.0	1.0	1.0	0.0	1.0	1.0	1.0	1.0	0.2	1.0	0.2	1.0	1.0	0.4	0	0	1
73	G	A	0.5	1.0	1.0	1.0	1.0	1.0	1.0	1.0	1.0	1.0	1.0	1.0	0.1	0.3	0.0	1.0	1.0	1.0	1.0	1.0	0	0	1
73	G	T	1.0	1.0	1.0	1.0	1.0	1.0	1.0	1.0	1.0	0.1	1.0	1.0	1.0	0.0	1.0	1.0	1.0	1.0	1.0	1.0	0	1	1
74	G	A	0.1	1.0	1.0	1.0	1.0	1.0	1.0	1.0	1.0	1.0	1.0	1.0	0.3	1.0	1.0	1.0	1.0	1.0	1.0	0.1	0	0	0
74	G	T	0.0	1.0	1.0	1.0	1.0	1.0	1.0	0.1	1.0	1.0	1.0	1.0	1.0	1.0	1.0	1.0	1.0	0.2	1.0	1.0	0	0	1
75	A	G	0.6	0.1	1.0	1.0	1.0	1.0	1.0	1.0	1.0	1.0	1.0	1.0	0.4	0.1	0.3	1.0	1.0	1.0	0.3	0.4	0	0	0
78	A	G	0.3	1.0	1.0	1.0	1.0	1.0	0.2	1.0	1.0	1.0	1.0	1.0	0.2	1.0	1.0	1.0	1.0	1.0	1.0	1.0	0	0	0
79	G	T	0.1	1.0	1.0	1.0	1.0	1.0	1.0	1.0	1.0	1.0	1.0	1.0	0.2	1.0	1.0	1.0	1.0	1.0	1.0	1.0	0	0	0
86	A	G	1.0	1.0	1.0	1.0	1.0	1.0	1.0	1.0	1.0	1.0	1.0	1.0	0.0	1.0	1.0	1.0	1.0	1.0	0.1	1.0	0	0	1
88	G	A	0.4	1.0	1.0	1.0	1.0	1.0	1.0	1.0	1.0	1.0	1.0	1.0	1.0	1.0	1.0	1.0	1.0	1.0	1.0	0.0	0	1	1
91	G	A	1.0	1.0	1.0	1.0	1.0	1.0	1.0	1.0	0.0	1.0	1.0	1.0	1.0	1.0	1.0	1.0	1.0	1.0	0.1	1.0	0	1	1
94	C	A	0.4	1.0	1.0	1.0	1.0	1.0	1.0	0.1	0.2	1.0	1.0	1.0	1.0	1.0	1.0	1.0	1.0	1.0	0.1	1.0	0	0	0
94	C	T	1.0	1.0	1.0	1.0	1.0	1.0	1.0	1.0	1.0	1.0	1.0	1.0	0.2	1.0	1.0	1.0	0.1	1.0	1.0	0.2	0	0	0
96	T	A	0.5	1.0	1.0	1.0	1.0	1.0	1.0	1.0	0.2	1.0	1.0	0.1	1.0	0.2	1.0	1.0	1.0	1.0	0.2	1.0	0	0	0
100	G	A	1.0	1.0	0.1	1.0	1.0	1.0	1.0	1.0	0.2	1.0	1.0	1.0	0.3	0.2	1.0	1.0	1.0	1.0	1.0	1.0	0	0	0
101	A	G	1.0	1.0	0.1	1.0	1.0	1.0	1.0	1.0	1.0	1.0	1.0	1.0	0.0	1.0	1.0	1.0	1.0	1.0	1.0	1.0	0	0	1
104	G	T	1.0	1.0	1.0	1.0	1.0	1.0	1.0	1.0	1.0	1.0	1.0	0.0	1.0	0.2	1.0	1.0	1.0	1.0	0.1	1.0	0	1	1
105	T	A	1.0	1.0	1.0	1.0	1.0	1.0	1.0	1.0	1.0	1.0	1.0	0.1	1.0	0.1	1.0	1.0	1.0	1.0	0.1	1.0	0	0	0
105	T	C	0.4	1.0	0.1	1.0	0.2	1.0	1.0	1.0	1.0	1.0	1.0	0.1	1.0	1.0	1.0	1.0	1.0	1.0	1.0	1.0	0	0	0
106	G	A	1.0	1.0	1.0	1.0	1.0	0.2	1.0	1.0	1.0	1.0	1.0	1.0	0.3	1.0	0.1	1.0	1.0	1.0	1.0	0.3	0	0	0
107	A	T	1.0	1.0	1.0	1.0	1.0	1.0	1.0	1.0	0.1	1.0	1.0	1.0	1.0	1.0	1.0	1.0	1.0	1.0	0.1	0.2	0	0	0
115	G	A	1.0	1.0	1.0	1.0	1.0	1.0	1.0	1.0	1.0	1.0	1.0	1.0	1.0	1.0	1.0	0.0	1.0	1.0	0.1	1.0	0	0	1
118	G	A	1.0	1.0	0.1	1.0	1.0	0.2	1.0	1.0	1.0	1.0	1.0	1.0	1.0	0.1	1.0	1.0	1.0	1.0	1.0	1.0	0	0	0
126	T	A	1.0	1.0	1.0	1.0	0.2	1.0	1.0	1.0	0.0	1.0	1.0	1.0	1.0	1.0	1.0	1.0	1.0	1.0	1.0	1.0	1	1	1
127	G	A	0.5	1.0	1.0	1.0	1.0	1.0	1.0	1.0	1.0	0.0	1.0	1.0	1.0	0.0	1.0	1.0	1.0	1.0	1.0	1.0	0	0	2
131	C	T	1.0	1.0	1.0	1.0	1.0	1.0	1.0	1.0	1.0	0.1	1.0	1.0	1.0	1.0	0.1	1.0	1.0	1.0	0.1	1.0	0	0	0
133	G	A	0.7	0.3	0.7	0.4	0.8	0.5	0.6	0.6	0.8	0.7	0.7	0.9	0.8	0.2	0.4	0.0	0.8	0.5	0.5	0.5	0	0	1
134	C	A	0.1	1.0	1.0	1.0	1.0	1.0	1.0	1.0	1.0	1.0	1.0	1.0	1.0	1.0	0.1	1.0	1.0	0.2	1.0	1.0	0	0	0
137	C	T	1.0	1.0	1.0	1.0	1.0	1.0	1.0	1.0	1.0	1.0	0.2	1.0	1.0	0.3	1.0	1.0	1.0	1.0	1.0	0.0	0	1	1
138	G	A	1.0	1.0	1.0	1.0	1.0	1.0	1.0	0.1	1.0	1.0	1.0	0.1	1.0	1.0	1.0	0.2	1.0	1.0	1.0	1.0	0	0	0
144	A	G	1.0	1.0	1.0	1.0	1.0	1.0	0.4	0.1	0.2	1.0	1.0	1.0	1.0	0.3	1.0	1.0	1.0	1.0	0.2	0.4	0	0	0
146	G	A	1.0	1.0	1.0	1.0	1.0	1.0	0.3	1.0	1.0	1.0	1.0	1.0	1.0	1.0	0.0	1.0	1.0	1.0	1.0	1.0	1	1	1
147	G	A	0.1	1.0	1.0	0.1	1.0	1.0	1.0	1.0	1.0	1.0	1.0	1.0	1.0	1.0	1.0	1.0	1.0	1.0	1.0	1.0	0	0	0
148	A	G	1.0	1.0	1.0	1.0	1.0	1.0	1.0	1.0	0.3	1.0	1.0	1.0	0.4	1.0	1.0	1.0	0.2	0.0	1.0	0.4	0	1	1
150	A	C	1.0	1.0	1.0	1.0	1.0	1.0	1.0	1.0	1.0	1.0	0.0	1.0	1.0	1.0	1.0	1.0	1.0	1.0	1.0	1.0	1	1	1
150	A	G	0.1	1.0	1.0	1.0	1.0	1.0	1.0	1.0	1.0	1.0	1.0	1.0	1.0	1.0	1.0	1.0	1.0	1.0	0.1	1.0	0	0	0
156	C	A	0.1	1.0	1.0	1.0	1.0	1.0	1.0	1.0	1.0	1.0	1.0	1.0	0.3	1.0	0.1	1.0	1.0	1.0	1.0	1.0	0	0	0
157	A	G	0.2	1.0	1.0	1.0	1.0	1.0	1.0	1.0	1.0	0.2	1.0	1.0	0.4	1.0	1.0	1.0	1.0	0.3	1.0	0.4	0	0	0
158	C	T	1.0	1.0	0.2	0.2	0.3	1.0	0.4	1.0	1.0	1.0	1.0	1.0	0.1	1.0	1.0	0.4	1.0	1.0	1.0	1.0	0	0	0
160	G	A	1.0	1.0	1.0	1.0	1.0	1.0	1.0	1.0	1.0	0.0	1.0	1.0	1.0	1.0	1.0	1.0	1.0	0.0	1.0	1.0	2	2	2
161	T	A	1.0	1.0	1.0	1.0	1.0	1.0	1.0	1.0	1.0	0.0	1.0	1.0	1.0	1.0	1.0	1.0	1.0	0.0	1.0	1.0	2	2	2
162	G	A	1.0	1.0	1.0	1.0	1.0	1.0	1.0	1.0	1.0	0.0	1.0	1.0	0.1	0.2	1.0	1.0	1.0	1.0	1.0	1.0	0	0	1
164	A	G	1.0	1.0	1.0	1.0	1.0	1.0	1.0	1.0	1.0	1.0	0.0	1.0	1.0	1.0	1.0	1.0	1.0	1.0	1.0	1.0	1	1	1
165	G	A	1.0	1.0	1.0	1.0	1.0	0.2	1.0	1.0	1.0	0.1	1.0	1.0	1.0	0.2	1.0	1.0	1.0	1.0	1.0	0.3	0	0	0
166	C	T	1.0	0.1	1.0	1.0	1.0	1.0	1.0	0.1	1.0	1.0	0.2	1.0	1.0	0.2	1.0	1.0	1.0	0.2	1.0	1.0	0	0	0
170	C	T	0.0	1.0	1.0	0.3	0.2	0.6	0.6	1.0	0.1	0.4	1.0	1.0	1.0	1.0	0.4	1.0	1.0	1.0	1.0	1.0	0	0	1
172	T	G	1.0	0.0	1.0	0.0	1.0	1.0	1.0	0.0	1.0	1.0	1.0	1.0	1.0	1.0	1.0	0.0	0.0	0.0	1.0	0.0	7	7	7
174	G	A	1.0	1.0	0.0	1.0	1.0	0.3	1.0	1.0	1.0	0.0	1.0	1.0	1.0	1.0	1.0	1.0	1.0	1.0	1.0	1.0	1	1	2
176	A	G	0.5	1.0	0.1	1.0	1.0	1.0	1.0	1.0	0.2	1.0	1.0	1.0	1.0	1.0	1.0	0.3	1.0	1.0	1.0	0.3	0	0	0
177	G	A	1.0	1.0	1.0	1.0	0.3	0.1	0.4	1.0	1.0	1.0	1.0	1.0	0.4	1.0	1.0	1.0	1.0	0.4	1.0	0.1	0	0	0
179	A	T	0.1	1.0	1.0	1.0	1.0	1.0	1.0	1.0	1.0	1.0	1.0	1.0	0.2	1.0	1.0	1.0	1.0	1.0	1.0	1.0	0	0	0
185	G	A	1.0	1.0	0.1	1.0	1.0	1.0	1.0	1.0	1.0	1.0	1.0	1.0	0.2	1.0	0.1	1.0	1.0	1.0	1.0	1.0	0	0	0
186	G	A	0.3	1.0	1.0	1.0	1.0	0.2	1.0	1.0	1.0	1.0	1.0	1.0	0.2	1.0	1.0	1.0	1.0	1.0	1.0	1.0	0	0	0
188	C	A	0.1	1.0	1.0	1.0	1.0	1.0	1.0	1.0	0.1	1.0	1.0	1.0	1.0	1.0	1.0	1.0	1.0	1.0	1.0	1.0	0	0	0
188	C	T	1.0	1.0	1.0	1.0	1.0	0.3	1.0	1.0	1.0	1.0	1.0	1.0	0.3	1.0	1.0	1.0	1.0	1.0	0.2	0.1	0	0	0
193	T	C	1.0	1.0	1.0	1.0	1.0	1.0	1.0	1.0	1.0	0.1	1.0	1.0	1.0	1.0	1.0	1.0	1.0	1.0	0.1	0.2	0	0	0
194	G	A	1.0	1.0	0.2	1.0	1.0	0.4	1.0	1.0	1.0	1.0	1.0	1.0	0.0	0.1	1.0	1.0	1.0	1.0	0.3	1.0	0	0	1
194	G	T	0.3	1.0	0.8	0.8	0.6	0.9	0.1	0.4	0.7	1.0	0.3	0.3	0.8	0.9	0.4	0.4	0.8	0.4	0.9	0.3	0	0	0
195	G	A	1.0	1.0	1.0	1.0	1.0	1.0	0.2	0.1	1.0	1.0	1.0	1.0	1.0	0.1	1.0	1.0	1.0	1.0	1.0	1.0	0	0	0
195	G	T	1.0	1.0	1.0	1.0	0.3	1.0	0.4	1.0	0.0	1.0	1.0	1.0	1.0	0.0	1.0	1.0	1.0	1.0	1.0	0.4	0	0	2
196	T	G	0.3	0.7	0.8	0.8	0.6	1.0	0.0	0.4	0.9	1.0	0.1	0.3	0.8	0.9	0.4	0.5	0.8	0.4	0.9	0.2	0	0	1
198	T	G	1.0	0.9	0.5	0.9	1.0	1.0	0.0	0.9	0.4	0.9	0.3	0.2	0.1	0.5	0.2	0.8	1.0	0.9	0.9	0.0	0	2	2
199	G	A	1.0	0.8	0.7	0.9	1.0	0.9	0.0	0.9	0.7	0.8	0.2	0.1	0.1	0.6	0.1	0.8	1.0	1.0	1.0	0.0	1	2	2
199	G	T	0.9	1.0	0.4	1.0	1.0	0.4	1.0	1.0	0.2	1.0	1.0	1.0	0.2	0.2	1.0	0.7	0.5	0.7	0.5	0.1	0	0	0
200	A	C	1.0	0.7	0.5	0.9	1.0	1.0	0.0	0.8	0.5	0.9	0.1	0.3	0.1	0.4	0.2	1.0	1.0	0.9	0.9	0.0	0	2	2

A. KeyPoint NVS analysis - amplicon 1

Observed counts per position per pool, when more than 2 polymorphisms per position

			Sums per pool																					
			###	627	832	955	###	###	###	738	###	###	###	###	###	###	###	###	###	###	###	###		
pos	WT	SNP	X1	X2	X3	X4	X5	X6	X7	X8	X9	X10	X11	X12	Y1	Y2	Y3	Y4	Y5	Y6	Y7	Y8	Total	error rate
200	A	G							1					1	1			1			1	1	6	0.00019
200	A	T					1		6					2	2		2	3				2	18	0.00057
201	T	C				1			1				1		2						2		7	0.00022
202	A	T				1			1				1		2			2			2	1	10	0.00032
203	A	G					1		1									1				3	6	0.00019
204	C	A			1				1			1		2	4	1		2			2		14	0.00044
204	C	T					1							1								1	3	0.00009
205	C	T																		3			3	0.00009
206	C	A	2								1												3	0.00009
206	C	T	1					1			1	1			2	1	1				1		9	0.00028
207	T	A						1			1	1			3	1	1			1			9	0.00028
207	T	C	2	1	1		4	1	4	3					2		2	2		2		1	25	0.00079
208	A	T	2	1	1		4	1	4	4					2		2	1		2		1	25	0.00079
209	C	A	2	1	1		4	1	4	4					2		2	1		2		1	25	0.00079
210	C	A	1			1	1		2				1		1							1	8	0.00025
210	C	T				87										123		1					211	0.00665
211	A	C					1		1									1				1	4	0.00013
212	C	A									2				1						1		4	0.00013
212	C	T						1	1					1	1								4	0.00013
213	T	C	1																		1	2	4	0.00013
216	A	T			1							1		1	2	1		2			1		9	0.00028
217	T	A	1		2							1		1	2	1		2			1		11	0.00035
220	C	A												1	1							2	4	0.00013
220	C	T													2							1	3	0.00009
221	G	A	2		1		1				2	5	2		1	1		1		1		1	18	0.00057
221	G	T	1							1								1					3	0.00009
222	A	G							1									3				1	5	0.00016
223	C	A	2				1		2			1											6	0.00019
223	C	T							1										1			1	3	0.00009
224	A	C	2				1		2	1		1											7	0.00022
224	A	G			1				2						1						1		5	0.00016
226	A	C	1				1						1			1		1					5	0.00016
227	C	T	1				1						1					1		1			5	0.00016
228	T	A	1				1						1					1					4	0.00013
229	G	A				1	1			1		1			1								5	0.00016
230	C	T	1		1		1				2		1							1			7	0.00022
231	T	G	1					1			2					1		1		1			7	0.00022
233	G	A	1					1	5	2		1			4		1	1				2	18	0.00057
233	G	C	2		1		2		1							1		1					8	0.00025
233	G	T	1			1		1			1			1	1	1	1	1		1			10	0.00032
236	G	A					1											1		1			3	0.00009
236	G	T	8		1	1		5			3	4	1	3	1	6	1	6	3	6	2	5	56	0.00177
239	G	A		1													1	1		1			4	0.00013
241	T	C									1		2										3	0.00009
Total			192	240	57	233	89	87	178	144	86	348	72	57	175	224	76	316	158	559	97	344	3732	

P-values per position per pool

														A	B	C	D	E	F	G	H		Count	
pos	WT	SNP	X1	X2	X3	X4	X5	X6	X7	X8	X9	X10	X11	X12	Y1	Y2	Y3	Y4	Y5	Y6	Y7	Y8	P<0.001	P<0.01	P<0.05
200	A	G	1.0	1.0	1.0	1.0	1.0	1.0	0.4	1.0	1.0	1.0	1.0	0.2	0.4	1.0	1.0	0.3	1.0	1.0	0.2	0.4	0	0	0
200	A	T	1.0	1.0	1.0	1.0	0.6	1.0	0.0	1.0	1.0	1.0	1.0	0.1	0.4	1.0	0.1	0.1	1.0	1.0	1.0	0.4	0	1	1
201	T	C	1.0	1.0	1.0	0.2	1.0	1.0	0.4	1.0	1.0	1.0	0.2	1.0	0.1	1.0	1.0	1.0	1.0	1.0	0.0	1.0	0	0	1
202	A	T	1.0	1.0	1.0	0.3	1.0	1.0	0.5	1.0	1.0	1.0	0.3	1.0	0.2	1.0	1.0	0.1	1.0	1.0	0.1	0.5	0	0	0
203	A	G	1.0	1.0	1.0	1.0	0.3	1.0	0.4	1.0	1.0	1.0	1.0	1.0	1.0	1.0	1.0	0.3	1.0	1.0	1.0	0.0	0	0	1
204	C	A	1.0	1.0	0.3	1.0	1.0	1.0	0.6	1.0	1.0	0.4	1.0	0.1	0.0	0.5	1.0	0.2	1.0	1.0	0.1	1.0	0	0	1
204	C	T	1.0	1.0	1.0	1.0	0.1	1.0	1.0	1.0	1.0	1.0	1.0	0.1	1.0	1.0	1.0	1.0	1.0	1.0	1.0	0.2	0	0	0
205	C	T	1.0	1.0	1.0	1.0	1.0	1.0	1.0	1.0	1.0	1.0	1.0	1.0	1.0	1.0	1.0	1.0	1.0	0.0	1.0	1.0	1	1	1
206	C	A	0.1	1.0	1.0	1.0	1.0	1.0	1.0	1.0	0.1	1.0	1.0	1.0	1.0	1.0	1.0	1.0	1.0	1.0	1.0	1.0	0	0	0
206	C	T	0.7	1.0	1.0	1.0	1.0	0.5	1.0	1.0	0.3	0.3	1.0	1.0	0.1	0.4	0.3	1.0	1.0	1.0	0.3	1.0	0	0	0
207	T	A	1.0	1.0	1.0	1.0	1.0	0.5	1.0	1.0	0.3	0.3	1.0	1.0	0.0	0.4	0.3	1.0	1.0	0.4	1.0	1.0	0	0	1
207	T	C	0.8	0.4	0.5	1.0	0.0	0.8	0.1	0.0	1.0	1.0	1.0	1.0	0.6	1.0	0.2	0.5	1.0	0.4	1.0	0.8	0	0	2
208	A	T	0.8	0.4	0.5	1.0	0.0	0.8	0.1	0.0	1.0	1.0	1.0	1.0	0.6	1.0	0.2	0.8	1.0	0.4	1.0	0.8	0	1	2
209	C	A	0.8	0.4	0.5	1.0	0.0	0.8	0.1	0.0	1.0	1.0	1.0	1.0	0.6	1.0	0.2	0.8	1.0	0.4	1.0	0.8	0	1	2
210	C	A	0.6	1.0	1.0	0.2	0.3	1.0	0.1	1.0	1.0	1.0	0.2	1.0	0.4	1.0	1.0	1.0	1.0	1.0	1.0	0.4	0	0	0
210	C	T	1.0	1.0	1.0	0.0	1.0	1.0	1.0	1.0	1.0	1.0	1.0	1.0	1.0	0.0	1.0	1.0	1.0	1.0	1.0	1.0	2	2	2
211	A	C	1.0	1.0	1.0	1.0	0.2	1.0	0.3	1.0	1.0	1.0	1.0	1.0	1.0	1.0	1.0	0.2	1.0	1.0	1.0	0.3	0	0	0
212	C	A	1.0	1.0	1.0	1.0	1.0	1.0	1.0	1.0	0.0	1.0	1.0	1.0	0.3	1.0	1.0	1.0	1.0	1.0	0.1	1.0	0	0	1
212	C	T	1.0	1.0	1.0	1.0	1.0	0.2	0.3	1.0	1.0	1.0	1.0	0.1	0.3	1.0	1.0	1.0	1.0	1.0	1.0	1.0	0	0	0
213	T	C	0.4	1.0	1.0	1.0	1.0	1.0	1.0	1.0	1.0	1.0	1.0	1.0	1.0	1.0	1.0	1.0	1.0	1.0	0.1	0.0	0	0	1
216	A	T	1.0	1.0	0.2	1.0	1.0	1.0	1.0	1.0	1.0	0.3	1.0	0.2	0.1	0.4	1.0	0.1	1.0	1.0	0.3	1.0	0	0	0
217	T	A	0.8	1.0	0.0	1.0	1.0	1.0	1.0	1.0	1.0	0.3	1.0	0.3	0.2	0.4	1.0	0.2	1.0	1.0	0.4	1.0	0	0	1
220	C	A	1.0	1.0	1.0	1.0	1.0	1.0	1.0	1.0	1.0	1.0	1.0	0.1	0.3	1.0	1.0	1.0	1.0	1.0	1.0	0.0	0	0	1
220	C	T	1.0	1.0	1.0	1.0	1.0	1.0	1.0	1.0	1.0	1.0	1.0	1.0	0.0	1.0	1.0	1.0	1.0	1.0	1.0	0.2	0	0	1
221	G	A	0.7	1.0	0.4	1.0	0.6	1.0	1.0	1.0	0.2	0.0	0.1	1.0	0.7	0.6	1.0	0.7	1.0	0.6	1.0	0.7	1	1	1
221	G	T	0.3	1.0	1.0	1.0	1.0	1.0	1.0	0.1	1.0	1.0	1.0	1.0	1.0	1.0	1.0	0.2	1.0	1.0	1.0	1.0	0	0	0
222	A	G	1.0	1.0	1.0	1.0	1.0	1.0	0.3	1.0	1.0	1.0	1.0	1.0	1.0	1.0	1.0	0.0	1.0	1.0	1.0	0.3	0	1	1
223	C	A	0.2	1.0	1.0	1.0	0.3	1.0	0.1	1.0	1.0	0.2	1.0	1.0	1.0	1.0	1.0	1.0	1.0	1.0	1.0	1.0	0	0	0
223	C	T	1.0	1.0	1.0	1.0	1.0	1.0	0.2	1.0	1.0	1.0	1.0	1.0	1.0	1.0	1.0	1.0	0.1	1.0	1.0	0.2	0	0	0
224	A	C	0.2	1.0	1.0	1.0	0.3	1.0	0.1	0.2	1.0	0.2	1.0	1.0	1.0	1.0	1.0	1.0	1.0	1.0	1.0	1.0	0	0	0
224	A	G	1.0	1.0	0.1	1.0	1.0	1.0	0.1	1.0	1.0	1.0	1.0	1.0	0.3	1.0	1.0	1.0	1.0	1.0	0.2	1.0	0	0	0
226	A	C	0.5	1.0	1.0	1.0	0.2	1.0	1.0	1.0	1.0	1.0	0.2	1.0	1.0	0.2	1.0	0.3	1.0	1.0	1.0	1.0	0	0	0
227	C	T	0.5	1.0	1.0	1.0	0.2	1.0	1.0	1.0	1.0	1.0	0.2	1.0	1.0	1.0	1.0	0.3	1.0	0.2	1.0	1.0	0	0	0
228	T	A	0.4	1.0	1.0	1.0	0.2	1.0	1.0	1.0	1.0	1.0	0.1	1.0	1.0	1.0	1.0	0.2	1.0	1.0	1.0	1.0	0	0	0
229	G	A	1.0	1.0	1.0	0.1	0.2	1.0	1.0	0.1	1.0	0.2	1.0	1.0	0.3	1.0	1.0	1.0	1.0	1.0	1.0	1.0	0	0	0
230	C	T	0.6	1.0	0.2	1.0	0.3	1.0	1.0	1.0	0.0	1.0	0.2	1.0	1.0	1.0	1.0	1.0	1.0	0.3	1.0	1.0	0	0	1
231	T	G	0.6	1.0	1.0	1.0	1.0	0.4	1.0	1.0	0.0	1.0	1.0	1.0	1.0	0.3	1.0	0.4	1.0	0.3	1.0	1.0	0	0	1
233	G	A	0.9	1.0	1.0	1.0	1.0	0.7	0.0	0.1	1.0	0.5	1.0	1.0	0.0	1.0	0.5	0.7	1.0	1.0	1.0	0.4	0	0	2
233	G	C	0.3	1.0	0.2	1.0	0.1	1.0	0.4	1.0	1.0	1.0	1.0	1.0	1.0	0.3	1.0	0.4	1.0	1.0	1.0	1.0	0	0	0
233	G	T	0.7	1.0	1.0	0.3	1.0	0.5	1.0	1.0	0.3	1.0	1.0	0.3	0.5	0.4	0.3	0.5	1.0	0.4	1.0	1.0	0	0	0
236	G	A	1.0	1.0	1.0	1.0	0.1	1.0	1.0	1.0	1.0	1.0	1.0	1.0	1.0	1.0	1.0	0.2	1.0	0.2	1.0	1.0	0	0	0
236	G	T	0.4	1.0	0.8	0.8	1.0	0.4	1.0	1.0	0.4	0.2	0.9	0.3	1.0	0.1	0.9	0.2	0.3	0.1	0.7	0.4	0	0	0
239	G	A	1.0	0.1	1.0	1.0	1.0	1.0	1.0	1.0	1.0	1.0	1.0	1.0	1.0	1.0	0.1	0.2	1.0	0.2	1.0	1.0	0	0	0
241	T	C	1.0	1.0	1.0	1.0	1.0	1.0	1.0	1.0	0.1	1.0	0.0	1.0	1.0	1.0	1.0	1.0	1.0	1.0	1.0	1.0	0	1	1

B. KeyPoint NVS analysis - amplicon 2

Observed counts per position per pool, when more than 2 polymorphisms per position

			Sums per pool																					
			342	283	652	175	270	890	373	###	933	495	658	692	341	274	636	412	832	864	###	57		
pos	WT	SNP	X1	X2	X3	X4	X5	X6	X7	X8	X9	X10	X11	X12	Y1	Y2	Y3	Y4	Y5	Y6	Y7	Y8	Total	error rate
44	G	A												1	1					1			3	0.00026
47	G	A			1				43		26		67		35			10		41			223	0.01940
65	C	T								1									1	1			3	0.00026
76	G	A																		2	2		4	0.00035
77	C	T			1			1										1		2	1		6	0.00052
101	G	A					1	2			2	1						3			2		11	0.00096
102	C	T			2		1		1								1		1	1	3		10	0.00087
125	A	G											1						2				3	0.00026
153	G	A					1	1					1	1				1	1				6	0.00052
157	G	A						1										1		1			3	0.00026
158	C	T			2			1				1									2		6	0.00052
165	C	T				1				2		1	1	1			2		2		1		11	0.00096
171	T	G				15										25							40	0.00348
174	G	A																1			2		3	0.00026
183	T	C										1								1	1		3	0.00026
185	C	T	1		1							1							1		1		5	0.00043
191	G	A	3				1						1			2				1			8	0.00070
192	C	T					2				1												3	0.00026
193	C	T				15		1								25							41	0.00357
203	A	C	1									75								69			145	0.01261
206	T	C													1			1		1			3	0.00026
209	T	C				15										25							40	0.00348
216	A	T						1					1								1		3	0.00026
223	T	C				1				3				4	1				1		2		12	0.00104
226	T	C	1	1					3	2	1	1	1	2	3		1				1		17	0.00148
227	C	T				1				3				4	1				1	1	2		13	0.00113
230	C	T	1				1	2	1	3	1		1	4	2		1			2	1		20	0.00174
233	T	C					1		1	2					1					1	1		7	0.00061
245	C	A				15										25	1						41	0.00357
245	C	T					2			2					1					1	1		7	0.00061
252	G	T						1						2									3	0.00026
253	T	G						1						2									3	0.00026
256	T	C									1		1				1						3	0.00026
266	A	G				7								1		12							20	0.00174
269	T	A		37		17				63								15	44	29		11	216	0.01879
283	G	A	2		1			1					1						1				6	0.00052
284	C	T			1						1								1				3	0.00026
329	C	A										2		1									3	0.00026
331	G	A					2				1												3	0.00026
343	C	T						2	1														3	0.00026
346	G	A											1							2			3	0.00026
Total			13	41	18	90	20	28	54	104	49	89	89	33	47	116	19	42	74	174	45	13	1158	

P-values per position per pool

														A	B	C	D	E	F	G	H		Count	
pos	WT	SNP	X1	X2	X3	X4	X5	X6	X7	X8	X9	X10	X11	X12	Y1	Y2	Y3	Y4	Y5	Y6	Y7	Y8	P<0.001	P<0.01	P<0.05
44	G	A	1.0	1.0	1.0	1.0	1.0	1.0	1.0	1.0	1.0	1.0	1.0	0.2	0.1	1.0	1.0	1.0	1.0	0.2	1.0	1.0	0	0	0
47	G	A	1.0	1.0	1.0	1.0	1.0	1.0	0.0	1.0	0.0	1.0	0.0	1.0	0.0	1.0	1.0	0.3	1.0	0.0	1.0	1.0	4	4	5
65	C	T	1.0	1.0	1.0	1.0	1.0	1.0	1.0	0.3	1.0	1.0	1.0	1.0	1.0	1.0	1.0	1.0	0.2	0.2	1.0	1.0	0	0	0
76	G	A	1.0	1.0	1.0	1.0	1.0	1.0	1.0	1.0	1.0	1.0	1.0	1.0	1.0	1.0	1.0	1.0	1.0	0.0	0.1	1.0	0	0	1
77	C	T	1.0	1.0	0.3	1.0	1.0	0.4	1.0	1.0	1.0	1.0	1.0	1.0	1.0	1.0	1.0	0.2	1.0	0.1	0.5	1.0	0	0	0
101	G	A	1.0	1.0	1.0	1.0	0.2	0.2	1.0	1.0	0.2	0.4	1.0	1.0	1.0	1.0	1.0	0.0	1.0	1.0	0.3	1.0	0	1	1
102	C	T	1.0	1.0	0.1	1.0	0.2	1.0	0.3	1.0	1.0	1.0	1.0	1.0	1.0	1.0	0.4	1.0	0.5	0.5	0.1	1.0	0	0	0
125	A	G	1.0	1.0	1.0	1.0	1.0	1.0	1.0	1.0	1.0	1.0	0.2	1.0	1.0	1.0	1.0	1.0	0.0	1.0	1.0	1.0	0	0	1
153	G	A	1.0	1.0	1.0	1.0	0.1	0.4	1.0	1.0	1.0	1.0	0.3	0.3	1.0	1.0	1.0	0.2	0.4	1.0	1.0	1.0	0	0	0
157	G	A	1.0	1.0	1.0	1.0	1.0	0.2	1.0	1.0	1.0	1.0	1.0	1.0	1.0	1.0	1.0	0.1	1.0	0.2	1.0	1.0	0	0	0
158	C	T	1.0	1.0	0.0	1.0	1.0	0.4	1.0	1.0	1.0	0.2	1.0	1.0	1.0	1.0	1.0	1.0	1.0	1.0	0.1	1.0	0	0	1
165	C	T	1.0	1.0	1.0	0.2	1.0	1.0	1.0	0.3	1.0	0.4	0.5	0.5	1.0	1.0	0.1	1.0	0.2	1.0	0.7	1.0	0	0	0
171	T	G	1.0	1.0	1.0	0.0	1.0	1.0	1.0	1.0	1.0	1.0	1.0	1.0	1.0	0.0	1.0	1.0	1.0	1.0	1.0	1.0	2	2	2
174	G	A	1.0	1.0	1.0	1.0	1.0	1.0	1.0	1.0	1.0	1.0	1.0	1.0	1.0	1.0	1.0	0.1	1.0	1.0	0.0	1.0	0	0	1
183	T	C	1.0	1.0	1.0	1.0	1.0	1.0	1.0	1.0	1.0	0.1	1.0	1.0	1.0	1.0	1.0	1.0	1.0	0.2	0.3	1.0	0	0	0
185	C	T	0.1	1.0	0.2	1.0	1.0	1.0	1.0	1.0	1.0	0.2	1.0	1.0	1.0	1.0	1.0	1.0	0.3	1.0	0.4	1.0	0	0	0
191	G	A	0.0	1.0	1.0	1.0	0.2	1.0	1.0	1.0	1.0	1.0	0.4	1.0	1.0	0.0	1.0	1.0	1.0	0.5	1.0	1.0	0	1	2
192	C	T	1.0	1.0	1.0	1.0	0.0	1.0	1.0	1.0	0.2	1.0	1.0	1.0	1.0	1.0	1.0	1.0	1.0	1.0	1.0	1.0	0	1	1
193	C	T	1.0	1.0	1.0	0.0	1.0	1.0	1.0	1.0	1.0	1.0	1.0	1.0	1.0	0.0	1.0	1.0	1.0	1.0	1.0	1.0	2	2	2
203	A	C	1.0	1.0	1.0	1.0	1.0	1.0	1.0	1.0	1.0	0.0	1.0	1.0	1.0	1.0	1.0	1.0	1.0	0.0	1.0	1.0	2	2	2
206	T	C	1.0	1.0	1.0	1.0	1.0	1.0	1.0	1.0	1.0	1.0	1.0	1.0	0.1	1.0	1.0	0.1	1.0	0.2	1.0	1.0	0	0	0
209	T	C	1.0	1.0	1.0	0.0	1.0	1.0	1.0	1.0	1.0	1.0	1.0	1.0	1.0	0.0	1.0	1.0	1.0	1.0	1.0	1.0	2	2	2
216	A	T	1.0	1.0	1.0	1.0	1.0	0.2	1.0	1.0	1.0	1.0	0.2	1.0	1.0	1.0	1.0	1.0	1.0	1.0	0.3	1.0	0	0	0
223	T	C	1.0	1.0	1.0	0.2	1.0	1.0	1.0	0.1	1.0	1.0	1.0	0.0	0.3	1.0	1.0	1.0	0.6	1.0	0.4	1.0	0	1	1
226	T	C	0.4	0.3	1.0	1.0	1.0	1.0	0.0	0.5	0.7	0.5	0.6	0.3	0.0	1.0	0.6	1.0	1.0	1.0	0.8	1.0	0	0	2
227	C	T	1.0	1.0	1.0	0.2	1.0	1.0	1.0	0.1	1.0	1.0	1.0	0.0	0.3	1.0	1.0	1.0	0.6	0.6	0.4	1.0	0	1	1
230	C	T	0.4	1.0	1.0	1.0	0.4	0.5	0.5	0.3	0.8	1.0	0.7	0.0	0.1	1.0	0.7	1.0	1.0	0.4	0.9	1.0	0	0	1
233	T	C	1.0	1.0	1.0	1.0	0.2	1.0	0.2	0.2	1.0	1.0	1.0	1.0	0.2	1.0	1.0	1.0	1.0	0.4	0.5	1.0	0	0	0
245	C	A	1.0	1.0	1.0	0.0	1.0	1.0	1.0	1.0	1.0	1.0	1.0	1.0	1.0	0.0	0.9	1.0	1.0	1.0	1.0	1.0	2	2	2
245	C	T	1.0	1.0	1.0	1.0	0.0	1.0	1.0	0.2	1.0	1.0	1.0	1.0	0.2	1.0	1.0	1.0	1.0	0.4	0.5	1.0	0	0	1
252	G	T	1.0	1.0	1.0	1.0	1.0	0.2	1.0	1.0	1.0	1.0	1.0	0.0	1.0	1.0	1.0	1.0	1.0	1.0	1.0	1.0	0	0	1
253	T	G	1.0	1.0	1.0	1.0	1.0	0.2	1.0	1.0	1.0	1.0	1.0	0.0	1.0	1.0	1.0	1.0	1.0	1.0	1.0	1.0	0	0	1
256	T	C	1.0	1.0	1.0	1.0	1.0	1.0	1.0	1.0	0.2	1.0	0.2	1.0	1.0	1.0	0.2	1.0	1.0	1.0	1.0	1.0	0	0	0
266	A	G	1.0	1.0	1.0	0.0	1.0	1.0	1.0	1.0	1.0	1.0	1.0	0.7	1.0	0.0	1.0	1.0	1.0	1.0	1.0	1.0	2	2	2
269	T	A	1.0	0.0	1.0	0.0	1.0	1.0	1.0	0.0	1.0	1.0	1.0	1.0	1.0	1.0	1.0	0.0	0.0	0.0	1.0	0.0	5	6	7
283	G	A	0.0	1.0	0.3	1.0	1.0	0.4	1.0	1.0	1.0	1.0	0.3	1.0	1.0	1.0	1.0	1.0	0.4	1.0	1.0	1.0	0	0	1
284	C	T	1.0	1.0	0.2	1.0	1.0	1.0	1.0	1.0	0.2	1.0	1.0	1.0	1.0	1.0	1.0	1.0	0.2	1.0	1.0	1.0	0	0	0
329	C	A	1.0	1.0	1.0	1.0	1.0	1.0	1.0	1.0	1.0	0.0	1.0	0.2	1.0	1.0	1.0	1.0	1.0	1.0	1.0	1.0	0	1	1
331	G	A	1.0	1.0	1.0	1.0	0.0	1.0	1.0	1.0	0.2	1.0	1.0	1.0	1.0	1.0	1.0	1.0	1.0	1.0	1.0	1.0	0	1	1
343	C	T	1.0	1.0	1.0	1.0	1.0	0.0	0.1	1.0	1.0	1.0	1.0	1.0	1.0	1.0	1.0	1.0	1.0	1.0	1.0	1.0	0	0	1
346	G	A	1.0	1.0	1.0	1.0	1.0	1.0	1.0	1.0	1.0	1.0	0.2	1.0	1.0	1.0	1.0	1.0	1.0	0.0	1.0	1.0	0	0	1

C. KeyPoint NVS analysis - amplicon 3

Observed counts per position per pool, when more than 2 polymorphisms per position

			X1	X2	X3	X4	X5	X6	X7	X8	X9	X10	X11	X12	Y1	Y2	Y3	Y4	Y5	Y6	Y7	Y8		
Sums per pool			###	###	###	###	912	508	###	###	###	###	###	###	###	###	751	###	###	665	###	###		
pos	WT	SNP	X1	X2	X3	X4	X5	X6	X7	X8	X9	X10	X11	X12	Y1	Y2	Y3	Y4	Y5	Y6	Y7	Y8	Total	error rate
30	C	T	1													1		1					3	0.00008
32	A	G								1	1		1							1			4	0.00011
33	G	A		1	1													1				1	4	0.00011
38	C	T									1	1									1		3	0.00008
40	G	A		1							1	1											3	0.00008
41	T	C			1	105										105							211	0.00564
42	G	A								1					1							1	3	0.00008
45	T	C				1						1			1								3	0.00008
46	C	A		1																	2		3	0.00008
46	C	T	1		1					3		1			1				2		2	1	12	0.00032
47	G	A									1				1				1			1	4	0.00011
48	G	A				2	1			1					1								5	0.00013
51	G	A								2			1										3	0.00008
51	G	T								1		1							1				3	0.00008
52	G	A	1	4	2				1	4	3	2	2		3	5	3	2	2			2	36	0.00096
54	T	A		1	1	1				6	1	1	1	1	2							2	17	0.00045
54	T	C	1		2					2	1	1	1	1				1				1	11	0.00029
54	T	G		1		1		2		2		2	2			1		3	1			2	17	0.00045
55	T	A	1			1				1						1						1	5	0.00013
55	T	C	3	1	3		2		1	8	1		1	2	3	2		5			1	2	35	0.00094
56	C	T				1			1	1			1	1	1								6	0.00016
63	C	T	1							2								1					4	0.00011
64	A	G		1						1		1						1	1			3	8	0.00021
65	C	T			1	1				1													3	0.00008
66	G	A								2		1	1		2							1	7	0.00019
67	T	C																1		1	1	2	5	0.00013
70	G	T	1											2									3	0.00008
78	G	A								3	2			1	1								7	0.00019
79	G	A		1												1				1			3	0.00008
84	C	T				108				1						111		1				1	222	0.00593
85	A	G		505	2	244	112		361	879	133	277	260	1	541	228		296	132	151		198	4320	0.11546
86	T	C								3													3	0.00008
87	A	G		1				1		1									1				4	0.00011
88	C	T	2			2	1			5			1	1									12	0.00032
89	G	A	1	3		1			2	4		1	1									1	14	0.00037
89	G	T	1							2													3	0.00008
90	C	T			1			1							1							1	4	0.00011
91	C	A	1	1																	1		3	0.00008
91	C	T		1		1									2								4	0.00011
92	T	C								2							1						3	0.00008
92	T	G									2		1								1		4	0.00011
98	T	C								2									1				3	0.00008
99	C	T				2				1									1				4	0.00011
101	G	A														1		1	1				3	0.00008
105	C	T			2				1	2	1												6	0.00016
107	C	A								1									1	1			3	0.00008
107	C	T								3													3	0.00008
108	T	C									1		2										3	0.00008
109	T	C	1							3				1	1								6	0.00016
110	C	A						4		1											1		6	0.00016
116	G	A	1							2					1			1				2	7	0.00019
120	G	A								2			1								1		4	0.00011
121	A	G			1					1					1							1	4	0.00011
122	C	T		1	1	1				1												2	6	0.00016
126	T	C							1	1									2		1		5	0.00013
130	G	T																			3		3	0.00008
131	A	G								4													4	0.00011
134	C	A									2							1					3	0.00008
134	C	T								1		1									1		3	0.00008
140	C	A								2												1	3	0.00008
145	G	A		1						2									1			1	5	0.00013
146	C	T	5	8	5		4	1	3	20	9	9	6	5	4	1	2	1	7	1	2	4	97	0.00259
147	A	C				1				1		1											3	0.00008
147	A	G	1		1	2	1	1	1	7	1		1	3					3		3	3	28	0.00075
147	A	T		2					1	3	2	1	3		1								13	0.00035
148	G	A	2	1						3													6	0.00016
148	G	T			1		1			1		3									2		8	0.00021
149	G	A			1															1		1	3	0.00008
150	A	G			3		1			4	1	2	1	2				1	1			1	17	0.00045
151	C	A	2												1								3	0.00008
151	C	T								2		2	1					3			1		9	0.00024
152	G	A			1		1			1	1				1			1			1		7	0.00019
155	G	A	1	4	1			1	1	14	4	1	1	1	2	2		1	1		2	2	39	0.00104
156	C	T	1		2					1					1					1			6	0.00016
159	A	T													1						1	1	3	0.00008
160	G	A								3													3	0.00008
162	G	A	1	1						2					2								6	0.00016
163	G	A			1											2							3	0.00008
164	C	T				1				2		3	1	2	1				1				11	0.00029
166	C	T		1	3	2																	6	0.00016
167	G	A									1							1			1	1	4	0.00011
169	C	T									1										2		3	0.00008
171	G	A																2				1	3	0.00008
171	G	T								3								3					6	0.00016
176	G	A	2			1				2		2			1							2	10	0.00027
176	G	T								1							2						3	0.00008
177	C	T	2	7	1	2			2	12	6	4	2	1	3	1		1	3		3	2	52	0.00139
185	C	A								1	1	1											3	0.00008
189	A	C																4					4	0.00011
Total			41	556	47	485	130	12	381	###	193	334	302	32	588	467	10	340	167	163	45	255	###	

P-values per position per pool

														A	B	C	D	E	F	G	H		Count	
pos	WT	SNP	X1	X2	X3	X4	X5	X6	X7	X8	X9	X10	X11	X12	Y1	Y2	Y3	Y4	Y5	Y6	Y7	Y8	P<0.001	P<0.01	P<0.05
30	C	T	0.1	1.0	1.0	1.0	1.0	1.0	1.0	1.0	1.0	1.0	1.0	1.0	1.0	0.1	1.0	0.2	1.0	1.0	1.0	1.0	0	0	0
32	A	G	1.0	1.0	1.0	1.0	1.0	1.0	1.0	0.5	0.2	1.0	0.2	1.0	1.0	1.0	1.0	1.0	1.0	0.1	1.0	1.0	0	0	0
33	G	A	1.0	0.1	0.2	1.0	1.0	1.0	1.0	1.0	1.0	1.0	1.0	1.0	1.0	1.0	1.0	0.2	1.0	1.0	1.0	0.2	0	0	0
38	C	T	1.0	1.0	1.0	1.0	1.0	1.0	1.0	1.0	0.2	0.1	1.0	1.0	1.0	1.0	1.0	1.0	1.0	1.0	0.1	1.0	0	0	0
40	G	A	1.0	0.1	1.0	1.0	1.0	1.0	1.0	1.0	0.2	0.1	1.0	1.0	1.0	1.0	1.0	1.0	1.0	1.0	1.0	1.0	0	0	0
41	T	C	1.0	1.0	1.0	0.0	1.0	1.0	1.0	1.0	1.0	1.0	1.0	1.0	1.0	0.0	1.0	1.0	1.0	1.0	1.0	1.0	2	2	2
42	G	A	1.0	1.0	1.0	1.0	1.0	1.0	1.0	0.4	1.0	1.0	1.0	1.0	0.2	1.0	1.0	1.0	1.0	1.0	1.0	0.2	0	0	0
45	T	C	1.0	1.0	1.0	0.1	1.0	1.0	1.0	1.0	1.0	0.1	1.0	1.0	0.2	1.0	1.0	1.0	1.0	1.0	1.0	1.0	0	0	0
46	C	A	1.0	0.1	1.0	1.0	1.0	1.0	1.0	1.0	1.0	1.0	1.0	1.0	1.0	1.0	1.0	1.0	1.0	1.0	0.0	1.0	0	0	1
46	C	T	0.5	1.0	0.4	1.0	1.0	1.0	1.0	0.4	1.0	0.5	1.0	1.0	0.6	1.0	1.0	1.0	0.1	1.0	0.1	0.6	0	0	0
47	G	A	1.0	1.0	1.0	1.0	1.0	1.0	1.0	1.0	0.2	1.0	1.0	1.0	0.2	1.0	1.0	1.0	0.1	1.0	1.0	0.2	0	0	0
48	G	A	1.0	1.0	1.0	0.0	0.1	1.0	1.0	0.6	1.0	1.0	1.0	1.0	0.3	1.0	1.0	1.0	1.0	1.0	1.0	1.0	0	1	1
51	G	A	1.0	1.0	1.0	1.0	1.0	1.0	1.0	0.1	1.0	1.0	0.1	1.0	1.0	1.0	1.0	1.0	1.0	1.0	1.0	1.0	0	0	0
51	G	T	1.0	1.0	1.0	1.0	1.0	1.0	1.0	0.4	1.0	0.1	1.0	1.0	1.0	1.0	1.0	1.0	0.1	1.0	1.0	1.0	0	0	0
52	G	A	0.9	0.1	0.5	1.0	1.0	1.0	0.8	0.9	0.4	0.6	0.5	1.0	0.5	0.0	0.0	0.6	0.4	1.0	1.0	0.7	0	0	2
54	T	A	1.0	0.5	0.6	0.4	1.0	1.0	1.0	0.1	0.7	0.6	0.6	0.5	0.3	1.0	1.0	1.0	1.0	1.0	1.0	0.3	0	0	0
54	T	C	0.4	1.0	0.1	1.0	1.0	1.0	1.0	0.6	0.5	0.4	0.4	0.3	1.0	1.0	1.0	0.5	1.0	1.0	1.0	0.5	0	0	0
54	T	G	1.0	0.5	1.0	0.4	1.0	0.0	1.0	0.8	1.0	0.2	0.2	1.0	1.0	0.5	1.0	0.1	0.5	1.0	1.0	0.3	0	0	1
55	T	A	0.2	1.0	1.0	0.1	1.0	1.0	1.0	0.6	1.0	1.0	1.0	1.0	1.0	0.2	1.0	1.0	1.0	1.0	1.0	0.3	0	0	0
55	T	C	0.3	0.8	0.2	1.0	0.2	1.0	0.7	0.4	0.9	1.0	0.8	0.4	0.4	0.4	1.0	0.1	1.0	1.0	0.8	0.7	0	0	0
56	C	T	1.0	1.0	1.0	0.2	1.0	1.0	0.2	0.7	1.0	1.0	0.2	0.2	0.3	1.0	1.0	1.0	1.0	1.0	1.0	1.0	0	0	0
63	C	T	0.2	1.0	1.0	1.0	1.0	1.0	1.0	0.2	1.0	1.0	1.0	1.0	1.0	1.0	1.0	0.2	1.0	1.0	1.0	1.0	0	0	0
64	A	G	1.0	0.3	1.0	1.0	1.0	1.0	1.0	0.8	1.0	0.3	1.0	1.0	1.0	1.0	1.0	0.4	0.3	1.0	1.0	0.0	0	0	1
65	C	T	1.0	1.0	0.1	0.1	1.0	1.0	1.0	0.4	1.0	1.0	1.0	1.0	1.0	1.0	1.0	1.0	1.0	1.0	1.0	1.0	0	0	0
66	G	A	1.0	1.0	1.0	1.0	1.0	1.0	1.0	0.4	1.0	0.3	0.3	1.0	0.1	1.0	1.0	1.0	1.0	1.0	1.0	0.4	0	0	0
67	T	C	1.0	1.0	1.0	1.0	1.0	1.0	1.0	1.0	1.0	1.0	1.0	1.0	1.0	1.0	1.0	0.2	1.0	0.1	0.2	0.0	0	0	1
70	G	T	0.1	1.0	1.0	1.0	1.0	1.0	1.0	1.0	1.0	1.0	1.0	0.0	1.0	1.0	1.0	1.0	1.0	1.0	1.0	1.0	0	1	1
78	G	A	1.0	1.0	1.0	1.0	1.0	1.0	1.0	0.2	0.1	1.0	1.0	0.2	0.4	1.0	1.0	1.0	1.0	1.0	1.0	1.0	0	0	0
79	G	A	1.0	0.1	1.0	1.0	1.0	1.0	1.0	1.0	1.0	1.0	1.0	1.0	1.0	0.1	1.0	1.0	1.0	0.1	1.0	1.0	0	0	0
84	C	T	1.0	1.0	1.0	0.0	1.0	1.0	1.0	1.0	1.0	1.0	1.0	1.0	1.0	0.0	1.0	1.0	1.0	1.0	1.0	1.0	2	2	2
85	A	G	1.0	0.0	1.0	0.0	0.0	1.0	0.0	0.0	0.0	0.0	0.0	1.0	0.0	0.0	1.0	0.0	0.0	0.0	1.0	0.0	14	14	14
86	T	C	1.0	1.0	1.0	1.0	1.0	1.0	1.0	0.0	1.0	1.0	1.0	1.0	1.0	1.0	1.0	1.0	1.0	1.0	1.0	1.0	0	0	1
87	A	G	1.0	0.1	1.0	1.0	1.0	0.1	1.0	0.5	1.0	1.0	1.0	1.0	1.0	1.0	1.0	1.0	0.1	1.0	1.0	1.0	0	0	0
88	C	T	0.1	1.0	1.0	0.0	0.3	1.0	1.0	0.1	1.0	1.0	0.4	0.4	1.0	1.0	1.0	1.0	1.0	1.0	1.0	1.0	0	0	1
89	G	A	0.5	0.0	1.0	0.3	1.0	1.0	0.1	0.3	1.0	0.5	0.5	1.0	1.0	1.0	1.0	1.0	1.0	1.0	1.0	0.6	0	0	1
89	G	T	0.1	1.0	1.0	1.0	1.0	1.0	1.0	0.1	1.0	1.0	1.0	1.0	1.0	1.0	1.0	1.0	1.0	1.0	1.0	1.0	0	0	0
90	C	T	1.0	1.0	0.2	1.0	1.0	0.1	1.0	1.0	1.0	1.0	1.0	1.0	0.2	1.0	1.0	1.0	1.0	1.0	1.0	0.2	0	0	0
91	C	A	0.1	0.1	1.0	1.0	1.0	1.0	1.0	1.0	1.0	1.0	1.0	1.0	1.0	1.0	1.0	1.0	1.0	1.0	0.1	1.0	0	0	0
91	C	T	1.0	0.1	1.0	0.1	1.0	1.0	1.0	1.0	1.0	1.0	1.0	1.0	0.0	1.0	1.0	1.0	1.0	1.0	1.0	1.0	0	0	1
92	T	C	1.0	1.0	1.0	1.0	1.0	1.0	1.0	0.1	1.0	1.0	1.0	1.0	1.0	1.0	0.1	1.0	1.0	1.0	1.0	1.0	0	0	0
92	T	G	1.0	1.0	1.0	1.0	1.0	1.0	1.0	1.0	0.0	1.0	0.2	1.0	1.0	1.0	1.0	1.0	1.0	1.0	0.2	1.0	0	0	1
98	T	C	1.0	1.0	1.0	1.0	1.0	1.0	1.0	0.1	1.0	1.0	1.0	1.0	1.0	1.0	1.0	1.0	0.1	1.0	1.0	1.0	0	0	0
99	C	T	1.0	1.0	1.0	0.0	1.0	1.0	1.0	0.5	1.0	1.0	1.0	1.0	1.0	1.0	1.0	1.0	0.1	1.0	1.0	1.0	0	1	1
101	G	A	1.0	1.0	1.0	1.0	1.0	1.0	1.0	1.0	1.0	1.0	1.0	1.0	1.0	0.1	1.0	0.2	0.1	1.0	1.0	1.0	0	0	0
105	C	T	1.0	1.0	0.0	1.0	1.0	1.0	0.2	0.3	0.3	1.0	1.0	1.0	1.0	1.0	1.0	1.0	1.0	1.0	1.0	1.0	0	0	1
107	C	A	1.0	1.0	1.0	1.0	1.0	1.0	1.0	0.4	1.0	1.0	1.0	1.0	1.0	1.0	1.0	1.0	0.1	0.1	1.0	1.0	0	0	0
107	C	T	1.0	1.0	1.0	1.0	1.0	1.0	1.0	0.0	1.0	1.0	1.0	1.0	1.0	1.0	1.0	1.0	1.0	1.0	1.0	1.0	0	0	1
108	T	C	1.0	1.0	1.0	1.0	1.0	1.0	1.0	1.0	0.2	1.0	0.0	1.0	1.0	1.0	1.0	1.0	1.0	1.0	1.0	1.0	0	1	1
109	T	C	0.3	1.0	1.0	1.0	1.0	1.0	1.0	0.1	1.0	1.0	1.0	0.2	0.3	1.0	1.0	1.0	1.0	1.0	1.0	1.0	0	0	0
110	C	A	1.0	1.0	1.0	1.0	1.0	0.0	1.0	0.7	1.0	1.0	1.0	1.0	1.0	1.0	1.0	1.0	1.0	1.0	0.3	1.0	1	1	1
116	G	A	0.3	1.0	1.0	1.0	1.0	1.0	1.0	0.4	1.0	1.0	1.0	1.0	0.4	1.0	1.0	0.3	1.0	1.0	1.0	0.1	0	0	0
120	G	A	1.0	1.0	1.0	1.0	1.0	1.0	1.0	0.2	1.0	1.0	0.2	1.0	1.0	1.0	1.0	1.0	1.0	1.0	0.2	1.0	0	0	0
121	A	G	1.0	1.0	0.2	1.0	1.0	1.0	1.0	0.5	1.0	1.0	1.0	1.0	0.2	1.0	1.0	1.0	1.0	1.0	1.0	0.2	0	0	0
122	C	T	1.0	0.2	0.2	0.2	1.0	1.0	1.0	0.7	1.0	1.0	1.0	1.0	1.0	1.0	1.0	1.0	1.0	1.0	1.0	0.1	0	0	0
126	T	C	1.0	1.0	1.0	1.0	1.0	1.0	0.2	0.6	1.0	1.0	1.0	1.0	1.0	1.0	1.0	1.0	0.0	1.0	0.2	1.0	0	0	1
130	G	T	1.0	1.0	1.0	1.0	1.0	1.0	1.0	1.0	1.0	1.0	1.0	1.0	1.0	1.0	1.0	1.0	1.0	1.0	0.0	1.0	1	1	1
131	A	G	1.0	1.0	1.0	1.0	1.0	1.0	1.0	0.0	1.0	1.0	1.0	1.0	1.0	1.0	1.0	1.0	1.0	1.0	1.0	1.0	0	1	1
134	C	A	1.0	1.0	1.0	1.0	1.0	1.0	1.0	1.0	0.0	1.0	1.0	1.0	1.0	1.0	1.0	0.2	1.0	1.0	1.0	1.0	0	0	1
134	C	T	1.0	1.0	1.0	1.0	1.0	1.0	1.0	0.4	1.0	0.1	1.0	1.0	1.0	1.0	1.0	1.0	1.0	1.0	0.1	1.0	0	0	0
140	C	A	1.0	1.0	1.0	1.0	1.0	1.0	1.0	0.1	1.0	1.0	1.0	1.0	1.0	1.0	1.0	1.0	1.0	1.0	1.0	0.2	0	0	0
145	G	A	1.0	0.2	1.0	1.0	1.0	1.0	1.0	0.2	1.0	1.0	1.0	1.0	1.0	1.0	1.0	1.0	0.2	1.0	1.0	0.3	0	0	0
146	C	T	0.6	0.0	0.5	1.0	0.2	0.7	0.7	0.4	0.2	0.1	0.3	0.3	0.9	1.0	0.6	1.0	0.1	0.8	1.0	0.9	0	0	1
147	A	C	1.0	1.0	1.0	0.1	1.0	1.0	1.0	0.4	1.0	0.1	1.0	1.0	1.0	1.0	1.0	1.0	1.0	1.0	1.0	1.0	0	0	0
147	A	G	0.8	1.0	0.7	0.2	0.5	0.3	0.7	0.3	0.9	1.0	0.7	0.1	1.0	1.0	1.0	1.0	0.1	1.0	0.2	0.3	0	0	0
147	A	T	1.0	0.1	1.0	1.0	1.0	1.0	0.4	0.5	0.2	0.5	0.0	1.0	0.6	1.0	1.0	1.0	1.0	1.0	1.0	1.0	0	0	1
148	G	A	0.0	0.2	1.0	1.0	1.0	1.0	1.0	0.1	1.0	1.0	1.0	1.0	1.0	1.0	1.0	1.0	1.0	1.0	1.0	1.0	0	0	1
148	G	T	1.0	1.0	0.3	1.0	0.2	1.0	1.0	0.8	1.0	0.0	1.0	1.0	1.0	1.0	1.0	1.0	1.0	1.0	0.1	1.0	0	1	1
149	G	A	1.0	1.0	0.1	1.0	1.0	1.0	1.0	1.0	1.0	1.0	1.0	1.0	1.0	1.0	1.0	1.0	1.0	0.1	1.0	0.2	0	0	0
150	A	G	1.0	1.0	0.0	1.0	0.3	1.0	1.0	0.4	0.7	0.2	0.6	0.1	1.0	1.0	1.0	0.6	0.5	1.0	1.0	0.7	0	0	1
151	C	A	0.0	1.0	1.0	1.0	1.0	1.0	1.0	1.0	1.0	1.0	1.0	1.0	0.2	1.0	1.0	1.0	1.0	1.0	1.0	1.0	0	0	1
151	C	T	1.0	1.0	1.0	1.0	1.0	1.0	1.0	0.5	1.0	0.1	0.3	1.0	1.0	1.0	1.0	0.0	1.0	1.0	0.4	1.0	0	0	1
152	G	A	1.0	1.0	0.3	1.0	0.2	1.0	1.0	0.7	0.4	1.0	1.0	1.0	0.4	1.0	1.0	0.3	1.0	1.0	0.3	1.0	0	0	0
155	G	A	0.9	0.1	0.8	1.0	1.0	0.4	0.8	0.0	0.3	0.9	0.8	0.8	0.8	0.4	1.0	0.9	0.8	1.0	0.6	0.7	0	0	1
156	C	T	0.3	1.0	0.0	1.0	1.0	1.0	1.0	0.7	1.0	1.0	1.0	1.0	0.3	1.0	1.0	1.0	1.0	0.1	1.0	1.0	0	0	1
159	A	T	1.0	1.0	1.0	1.0	1.0	1.0	1.0	1.0	1.0	1.0	1.0	1.0	0.2	1.0	1.0	1.0	1.0	1.0	0.1	0.2	0	0	0
160	G	A	1.0	1.0	1.0	1.0	1.0	1.0	1.0	0.0	1.0	1.0	1.0	1.0	1.0	1.0	1.0	1.0	1.0	1.0	1.0	1.0	0	0	1
162	G	A	0.3	0.2	1.0	1.0	1.0	1.0	1.0	0.3	1.0	1.0	1.0	1.0	0.1	1.0	1.0	1.0	1.0	1.0	1.0	1.0	0	0	0
163	G	A	1.0	1.0	0.1	1.0	1.0	1.0	1.0	1.0	1.0	1.0	1.0	1.0	1.0	0.0	1.0	1.0	1.0	1.0	1.0	1.0	0	1	1
164	C	T	1.0	1.0	1.0	0.3	1.0	1.0	1.0	0.6	1.0	0.0	0.4	0.1	0.5	1.0	1.0	1.0	0.3	1.0	1.0	1.0	0	0	1
166	C	T	1.0	0.2	0.0	0.0	1.0	1.0	1.0	1.0	1.0	1.0	1.0	1.0	1.0	1.0	1.0	1.0	1.0	1.0	1.0	1.0	0	1	2
167	G	A	1.0	1.0	1.0	1.0	1.0	1.0	1.0	1.0	0.2	1.0	1.0	1.0	1.0	1.0	1.0	0.2	1.0	1.0	0.2	0.2	0	0	0
169	C	T	1.0	1.0	1.0	1.0	1.0	1.0	1.0	1.0	0.2	1.0	1.0	1.0	1.0	1.0	1.0	1.0	1.0	1.0	0.0	1.0	0	0	1
171	G	A	1.0	1.0	1.0	1.0	1.0	1.0	1.0	1.0	1.0	1.0	1.0	1.0	1.0	1.0	1.0	0.0	1.0	1.0	1.0	0.2	0	0	1
171	G	T	1.0	1.0	1.0	1.0	1.0	1.0	1.0	0.1	1.0	1.0	1.0	1.0	1.0	1.0	1.0	0.0	1.0	1.0	1.0	1.0	0	1	1
176	G	A	0.1	1.0	1.0	0.3	1.0	1.0	1.0	0.6	1.0	0.1	1.0	1.0	0.5	1.0	1.0	1.0	1.0	1.0	1.0	0.1	0	0	0
176	G	T	1.0	1.0	1.0	1.0	1.0	1.0	1.0	0.4	1.0	1.0	1.0	1.0	1.0	1.0	0.0	1.0	1.0	1.0	1.0	1.0	0	1	1
177	C	T	0.8	0.0	0.9	0.4	1.0	1.0	0.6	0.3	0.2	0.3	0.7	0.9	0.7	0.8	1.0	0.9	0.3	1.0	0.5	0.9	0	1	1
185	C	A	1.0	1.0	1.0	1.0	1.0	1.0	1.0	0.4	0.2	0.1	1.0	1.0	1.0	1.0	1.0	1.0	1.0	1.0	1.0	1.0	0	0	0
189	A	C	1.0	1.0	1.0	1.0	1.0	1.0	1.0	1.0	1.0	1.0	1.0	1.0	1.0	1.0	1.0	0.0	1.0	1.0	1.0	1.0	1	1	1
